# Role of dynamic nuclear deformation on genomic architecture reorganization

**DOI:** 10.1371/journal.pcbi.1007289

**Published:** 2019-09-11

**Authors:** Sungrim Seirin-Lee, Fumitaka Osakada, Junichi Takeda, Satoshi Tashiro, Ryo Kobayashi, Takashi Yamamoto, Hiroshi Ochiai

**Affiliations:** 1 Department of Mathematics, School of Science, Hiroshima University, Higashi-Hiroshima, Japan; 2 PRESTO, Japan Science and Technology Agency, Kawaguchi, Japan; 3 Laboratory of Cellular Pharmacology, Graduate School of Pharmaceutical Sciences, Nagoya University, Nagoya, Japan; 4 Department of Cellular Biology, Research Institute for Radiation Biology and Medicine, Hiroshima University, Hiroshima, Japan; 5 Department of Mathematical and Life Sciences, Graduate School of Science, Hiroshima University, Higashi-Hiroshima, Japan; Rutgers University, UNITED STATES

## Abstract

Higher-order genomic architecture varies according to cell type and changes dramatically during differentiation. One of the remarkable examples of spatial genomic reorganization is the rod photoreceptor cell differentiation in nocturnal mammals. The inverted nuclear architecture found in adult mouse rod cells is formed through the reorganization of the conventional architecture during terminal differentiation. However, the mechanisms underlying these changes remain largely unknown. Here, we found that the dynamic deformation of nuclei via actomyosin-mediated contractility contributes to chromocenter clustering and promotes genomic architecture reorganization during differentiation by conducting an *in cellulo* experiment coupled with phase-field modeling. Similar patterns of dynamic deformation of the nucleus and a concomitant migration of the nuclear content were also observed in rod cells derived from the developing mouse retina. These results indicate that the common phenomenon of dynamic nuclear deformation, which accompanies dynamic cell behavior, can be a universal mechanism for spatiotemporal genomic reorganization.

## Author summary

The motion and spatial reorganization of sub-nuclear domains have been extensively studied using microscopy, but the underlying mechanisms that promote the reorganization are still poorly understood. We found that dynamic nuclear deformation provides a driving force for long-range migration and aggressive clustering of chromocenters, which ultimately induces nuclear architecture reorganization. This study significantly contributes to an improved understanding of the role of nuclear deformation and reorganization of nuclear architecture that seems complex but is based on simple physical properties of the cell.

## Introduction

**Fig 1:**
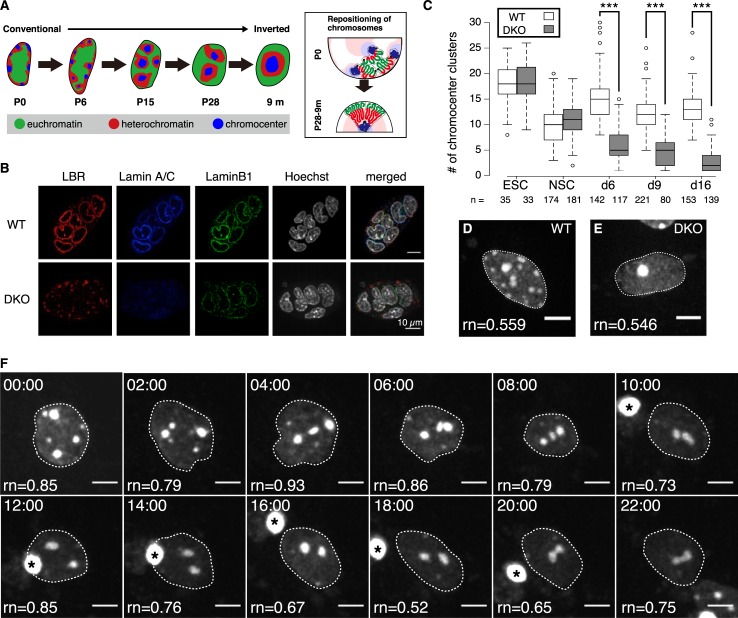
Experimental model cell system for nuclear architecture reorganization. (**A**) Mouse rod photoreceptor cells at 0, 6, 15 and 28 days after birth and 9 months after birth (P6, P15, P28, and 9 m respectively). The right panel diagram shows chromosome repositioning during the nuclear architecture reorganization. (**B**) An immunofluorescence assay showing expression of LBR, LamA/C, and lamin B1 in wild-type (WT) and double-knockout (DKO) cells. Scale bar: 10 μm. (**C**) The number of chromocenter (CC) clusters during differentiation. *, statistical difference by the Student's *t*-test: ***P < 10^−10^. (**D, E**) Fluorescence images of the nuclei of WT and DKO cells at 16 days post-differentiation. The values of roundness (rn) of each nuclei are shown. (**F**) Time-lapse microscopy of DKO cells 3 days post-differentiation at 30 min intervals for 44 h (S1–S3 Movies). Asterisks represent cellular debris. The values of roundness (rn) of each time frame are shown. The dotted curves in panels (D–E), (F) identify the nuclear edge. Scale bars: 5 μm.

In a typical nucleus during interphase, the long strands of genomic DNA are organized in a cell-type specific spatial architecture that plays an important role in DNA transcription, repair, and replication [1]. This architecture is partly determined by chromatin loops that form a higher-order genomic structure and heterochromatin “anchors”, including the lamin B receptor (LBR) and lamins A/C (LamA/C) [2], situated in the nuclear envelope [3–5]. The localization of heterochromatin at the nuclear periphery, the so-called conventional nuclear architecture, has been observed in most cell types of multicellular organisms. During cell differentiation, the nuclear architecture undergoes a reorganization process. Although most regions of the genome show no detectable reorganization from stage to stage, the ones reorganizing are enriched in genes related to the particular developmental programming. One of the most extreme examples of this genomic reorganization is the one occurring during the differentiation of rod photoreceptor cells found in nocturnal mammals, including mice [6]. In the cell precursors of rod photoreceptors, heterochromatin and the chromocenters (CCs, mainly consisting of highly-condensed centromere heterochromatic regions) are located at the nuclear periphery, whereas euchromatin is localized deeper inside the nucleus. This nuclear conformation becomes inverted during differentiation into mature rod photoreceptor cells. Moreover, there is a reduction in the nuclear volume as well as deformation, going from an ellipsoidal to a spherical shape, over time scales of several weeks [6]. Specifically, the CCs gather into a single cluster at the nuclear center and are surrounded by heterochromatin, while euchromatin shifts to the nuclear periphery (Fig 1A).

This inverted nuclear architecture appears to be unique to rod photoreceptor cells, olfactory and striatal neurons [7,8], and has not been observed in any other mouse tissues. It has been supposed that the inverted architecture is more advantageous to perceive light than the conventional architecture. Thus, the inverted architecture is likely to be commonly observed in rod photoreceptor cells of nocturnal mammals [6]. Mouse rod precursor cells express LBR, but not LamA/C, and the loss of LBR during differentiation is believed to trigger the reorganization into the inverted architecture [2]. Recent studies have shown that heterochromatin protein 1 (HP1), which localizes within CCs, isolates individual CC clusters within the nuclear space by phase separation until they eventually merge [9,10], suggesting that CCs can fuse when they are in close contact with each other. However, since genomic DNA molecules are long string-like polymers, individual genomic loci and sub-nuclear compartments can only diffuse in a limited space that is much smaller than the nuclear volume [11]. This implies that spatiotemporal reorganization of the nuclear architecture cannot be simply explained by the diffusion of sub-nuclear compartments.

On one hand, a theoretical study using a phase-field model suggested that the nuclear shape could affect both the spatial and temporal dynamics of nuclear architecture reorganization during the differentiation of mouse rod photoreceptor cells. However, this has not been proved experimentally [12]. In other words, this suggests that if an unknown mechanism other than diffusion plays a driving role in nuclear architecture reorganization, nuclear deformation may play a role as an external force.

Nuclear deformation is closely related to cell movement and shape. This is because the cytoskeleton, including actin fibers and microtubules, is connected to lamins, which interact with membrane-associated proteins located within the nuclear membrane to form the nuclear lamina, via the Linker of Nucleoskeleton and Cytoskeleton (LINC) complex on the nuclear envelope [13]. Cells detect mechanical force from the extracellular environment and as a response control the gene expression program by transmitting signals to the nucleus via the LINC complex [14]. However, it is not well understood whether deformation of the nucleus driven by the cytoskeleton causes the dramatic nuclear architecture reorganization as observed in the developing mouse retina.

Here, using interdisciplinary approaches including *in cellulo* and *ex vivo* experiments, along with mathematical modeling, we found that the “dynamic” nuclear deformation via actomyosin-mediated contractility contributes to the long-range migration of CCs and can induce the reorganization of the nuclear architecture. Our results suggest that dynamic nuclear deformation plays a critical role as a driving force inducing genomic architecture reorganization and may be a universal mechanism for spatiotemporal genomic reorganization during differentiation.

## Results

### CC migration is concomitant with dynamic nuclear deformation

Heterochromatin and CC are tethered by LBR and/or LamA/C protein localized at the nuclear membrane [2]. Basically, heterochromatin surrounds CC (Fig 1A). Since most tissues express LBR and/or LamA/C, they display the conventional nuclear architecture, in which heterochromatin and CC localize at the nuclear periphery. On the other hand, most cell types in *LBR*/*LamA* double-knockout (DKO) mouse show inverted nuclear architecture [2].

To address the mechanisms involved in CC clustering and nuclear architecture reorganization, we used mouse embryonic stem cells (mESCs) as an experimental model that can be genetically modified and differentiated into other cell types easily. More specifically, we used chromocenter clustering as a proxy measure for both CC and heterochromatin configuration. *LBR*/*LamA* DKO mESCs were generated using a CRISPR-Cas9 system [15–17] (Fig 1B and S1 Fig–S5 Fig). The number of CC clusters detected in DKO mESCs was comparable to that in the wild-type (WT) mESCs (Fig 1C). Inverted nuclear architecture has only been observed in terminally differentiated cells in prenatal DKO mice [2], suggesting that conversion into the inverted nuclear architecture only occurs upon termination of cell division. For this purpose, the DKO and WT mESCs were induced to differentiate into neural stem cells (NSCs), and then into post-mitotic neurons (S2 Fig–S5 Fig). During differentiation, the number of CC clusters steadily decreased in DKO cells (Fig 1C–1E, S6 Fig–S8 Fig). Furthermore, we confirmed that exogenous LBR expression prevented CC clustering in DKO cells (S7 Fig). Our data suggest that this model cell system recapitulates CC clustering, as observed during differentiation of rod photoreceptor cells.

To examine the nuclear dynamics of CCs, we performed live-cell imaging of the DKO NSCs after their differentiation into post-mitotic neurons. Three days after differentiation, cell nuclei were stained with SiR-Hoechst to visualize the DNA [18], followed by live-cell imaging with 30 min intervals for 44 hours (Fig 1F). We observed continuous nuclear deformation with concomitant movement of CCs and CC clustering (Fig 1F and S1 Movie to S3 Movie). Given these observations, we speculated that this “dynamic” nuclear deformation could potentially provide a driving force for CC clustering with a fast time scale.

### Dynamic nuclear deformation can induce the reorganization of the nuclear architecture

**Fig 2:**
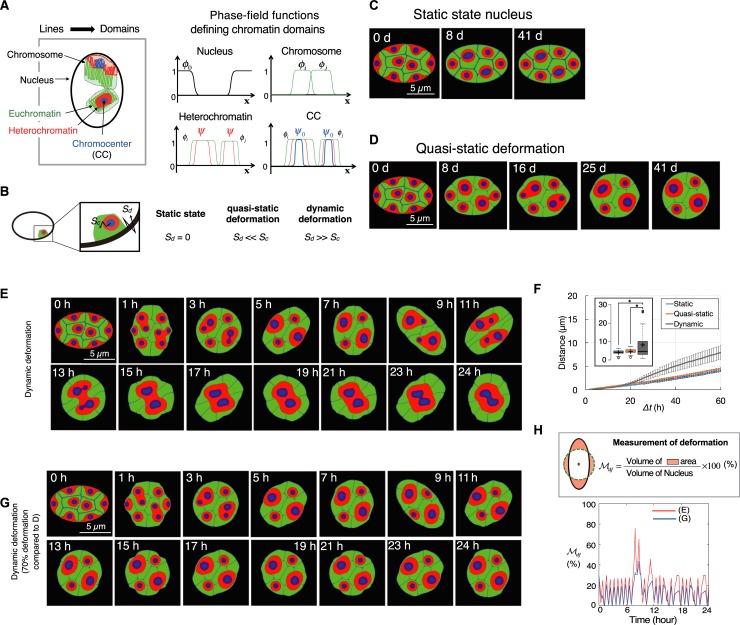
Mathematical modeling of nuclear deformation and architecture reorganization. (**A**) Domains representing the nucleus (black outline) and a chromosome containing euchromatin (green), heterochromatin (red) and a chromocenter (CC, blue) are described using phase-field functions with two stable states (0 and 1). (**B**) Schematic representation of speeds of nuclear deformation (*S*_*d*_) and the sub-nuclear compartment (*S*_*c*_). Color codes of sub-nuclear compartments are the same as in (A). (**C-E**) Simulation results of nuclei with (**C**) static state, (**D**) quasi-static and (**E**) dynamic deformation. (**F**) The averaged distance between positions of a CC cluster at time *t* and given reference position (*t = 0*) in static, quasi-static, and dynamically deforming nucleus (N > 30). The solid lines and error bars represent the average and SEM, respectively. Inset represents boxplots of the data at *Δt* = 60. *, statistical difference by the Student's *t*-test (*P* < 0.01). (**G**) Simulation results of nuclei with dynamic deformation with approximately 30% decreased deformability (i.e. 70% deformation) compared to (**E**) (See Material and Method for more detail). (**H**) The deformation degrees for (**E**) and (**G**), measured by means of the deformation-related area from a circle of the same area.

To investigate whether dynamic nuclear deformation can drive CC clustering and whether it has an effect on the nuclear architecture reorganization, we developed a mathematical model considering sub-nuclear domains, including euchromatin, heterochromatin, and CCs, as domains with territory (Fig 2A, S9 Fig and S10 Fig and Material and Method) [12]. The model was described by linking the dynamics of sub-nuclear compartments to domains using phase-field functions and developed by formulating interface properties of the sub-nuclear domain functions without taking into account the molecular details of DNA (Fig 2A). Thus, the total migration effect due to diffusion of each DNA molecule was coarse-grained as the macro-dynamics of sub-nuclear domains. We defined the nucleus, *ϕ*_0_(**x**,*t*); chromosome territories, *ϕ*_*m*_(**x**,*t*) (1≤*m*≤*N*); heterochromatin, *ψ*(**x**,*t*); and chromocenters (CCs), *ψ*_0_(**x**,*t*); where **x**∈*Ω* in **R**^2^ and *t*>0. *N* represents the total number of chromosomes in the nucleus, and we chose *N* = 8 for representative simulations. Based on the multi-phase-field modeling method, the sub-nuclear compartments model is given to:

$$\begin{array}{lll} & & \frac{\partial \phi_{m}}{\partial t}=\varepsilon_{\phi }^{2}\nabla^{2}\phi_{m}+\phi_{m}(1-\phi_{m})\left[\phi_{m}-\frac{1}{2}-A_{m}\phi_{m}(1-\phi_{m})\right],\;(1\leq m\leq N)\\ & & \frac{\partial \psi }{\partial t}=\varepsilon_{\psi }^{2}\nabla^{2}\psi +\psi (1-\psi )\left[\psi -\frac{1}{2}-B\psi (1-\psi )\right],\\ & & \frac{\partial \psi_{0}}{\partial t}=\varepsilon_{\psi_{0}}^{2}\nabla^{2}\psi_{0}+\psi_{0}(1-\psi_{0})\left[\psi_{0}-\frac{1}{2}-B_{0}\psi_{0}(1-\psi_{0})\right]. \end{array}$$

where *A*_*m*_, *B* and *B*_0_ are functions determined by the following assumptions: (i) every individual chromosome constitute a separate territory [19], (ii) the volume of each domain does not change over time and the nuclear space is fully occupied by chromosomes, and (iii) CC domains (or heterochromatin domains) can be fused when they are in contact with each other (S9B and S9C Fig) [6,9,10]. These modeling assumptions ensure that there is no long-range attraction force between CC clusters and that the fusion of CC clusters only occurs when they are closely positioned. Note that the assumption (ii) is only applied to our representative simulations to show the deformation effect by nuclear shapes. In general assumptions of our model, the volume is given to temporally varied values which are relatively determined by nuclear size at every given time (See Material and Method, Model development and The effect of nuclear size on nuclear architecture reorganization in more detail).

To examine the effect of nuclear deformation on CC clustering, we explicitly defined a phase-field function describing the nucleus and modeled the interface that was temporally and randomly changed, independently of the internal chromatin dynamics. That is, we directly controlled the nuclear deformation using the following equation:

$$\mu^{-1}\frac{\partial \phi_{0}}{\partial t}=\epsilon_{0}^{2}\nabla^{2}\phi_{0}+\phi_{0}(1-\phi_{0})\left[\phi_{0}-\frac{1}{2}-D(x,t)\phi_{0}(1-\phi_{0})\right],$$

where D(x,t) is a deformation function, which controls the interface movement of the nuclear phase-field (described in Material and Method). We first classified the nuclear deformation as a “static state” (un-deformed), “quasi-static deformation”, and “dynamic deformation” by varying the time scale and speed of nuclear deformation (See Material and Method for details; Fig 2B). Quasi-static nuclear deformation is defined as the situation when the speed of nuclear deformation (*S*_*d*_) is much slower than the movement speed of the sub-nuclear compartment (*S*_*c*_) and when the time scale of the nuclear deformation is similar to the one that has been supposed in a mouse rod photoreceptor cell (change in the nucleus morphology from an ellipsoidal to spherical shape over time scales of several weeks) (Fig 2B). In contrast, dynamic deformation is defined as the situation when the speed of nuclear deformation is much faster than the movement speed of the sub-nuclear compartment. The static state is defined as the situation when there is no nuclear deformation (*S*_*d*_ = 0). The static state is an unnatural and hypothetical state because cells fundamentally behave dynamically.

Using this mathematical model, we explored the three states of nuclear deformation. In both the static state and quasi-static nucleus states, partial clustering was observed but complete CC clustering could not be achieved spontaneously (Fig 2C and 2D), although the quasi-static nucleus showed a similar physiological deformation condition to the one that had been supposed [6]. However, dynamic nuclear deformation could accelerate the migration of CCs over a faster time scale than the others, resulting in complete CC clustering (Fig 2E and S4 Movie). Dynamic deformation strongly affected the relocation and shape of chromosome territories, and ultimately induced a long-range movement and the complete clustering of CC confined within each chromosome. We also measured the distances of CC movements during simulations of static and quasi-static nuclei and dynamically deforming nucleus and confirmed that dynamic nuclear deformation induces a significantly longer range of CC migration than the static state case (Fig 2F). This result suggests that if the nuclear deformation is a driving force of CC migration, CC clustering could be impaired by the attenuation of nuclear deformation. Indeed, when we reduced the degree of nuclear deformation by approximately 30%, CC clustering was partially impaired (Fig 2G and 2H).

We also tested effects of the nuclear size decrease on the CC clustering in simulations because it has been reported that reduction in nuclear size as well as nuclear morphology changes were observed during the nuclear architecture reorganization process in mouse rod photoreceptor cells [6]. Similarly, nuclear size reduction was also observed in our *in cellulo* model systems (S6 Fig). However, the mathematical model test did not show significant effects of the nuclear size decrease on CC clustering (S10 Fig, Material and Method, The effect of nuclear size on nuclear architecture reorganization). We further found that the timescale for CC clustering can be varied from several hours to several days due to the randomness of deformation direction and degree in our numerical simulation (see Fig 2D and 2E as examples). Consistent with this, in our *in cellulo* model systems, about half of the cells showed complete CC clustering at 16 days after differentiation induction, whereas the rest of them showed still multiple CC clusters with some variation (S11 Fig). This indicates that the variation of the number of CC clusters and timescale in the *in cellulo* experiment may be caused by the randomness of dynamic deformation. Taken together, our mathematical model suggests that dynamic nuclear deformation plays a role in inducing the long-range migration of CCs, which can result in a reorganization of the nuclear architecture with a fast time scale.

### Suppression of nuclear deformation results in impairment of CC clustering in a model cell system

Based on the mathematical modeling results, we next used our model cell system to explore whether suppression of nuclear deformation would affect the efficiency of CC clustering. Nuclear deformation is closely related to cell movement. This is because the cytoskeleton, including actin and microtubules, are connected to lamins via LINC complexes in the nuclear envelope [13]. Recently, it was reported that cytoskeletal forces can affect nuclear deformation and CC mobility [20]. Thus, to determine whether cytoskeletal forces affect the degree of nuclear deformation, CC mobility and clustering, DKO cells were treated with an inhibitor of either dynein, microtubule depolymerization, microtubule polymerization, myosin II or actin polymerization and monitored by live imaging.

**Fig 3:**
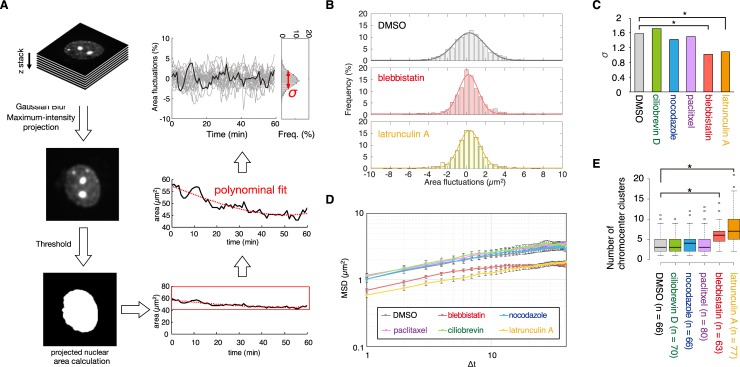
Effect of inhibition of dynamic nuclear deformation on CC clustering. (**A**) Schematic representation of quantification of projected nuclear-area fluctuation. (**B**) Normalized histograms of the combined projected nuclear-area fluctuations for all the cells and time points in DKO cells treated with either DMSO (control), blebbistatin or latrunculin A (S12 Fig). The solid curves represent Gaussian fits. (**C**) Standard deviations of the distributions of the nuclear-area fluctuation in cells treated with various drugs or the DMSO control (S12 Fig). Two-sample F-tests were done to assess significance; * denotes *P* < 0.01. (**D**) Ensemble- and time-averaged mean square displacement (MSD) curves between CC clusters and the nuclear center of mass in cells treated with the various drugs or the DMSO control during the 1 hour imaging period (S13A Fig). Error bars represent SEM. (**E**) Quantification of CC clusters observed during differentiation following drug treatment.

To quantify nuclear deformation, we calculated the projected nuclear-area fluctuations from the live images as described previously (Fig 3A) [20]. Briefly, the projected nuclear-area was measured by thresholding a maximum intensity projection of z-stacks of the nucleus. This projected area was then plotted as a function of time and fitted with third-order polynomial curves. The residual values were divided by the value of the polynomial at each time point and multiplied by 100 to calculate normalized residual area fluctuations as percentages. Such fluctuation values were combined from multiple cells and time points for each condition to obtain a normal distribution. Standard deviation (σ) of such a distribution indicates the amplitude of area fluctuations (Fig 3A). To quantify CC mobility, we calculated the mean squared displacement of the center of mass of CCs relative to the nuclear center of mass from the live images. We found that the myosin II inhibitor (blebbistatin) and actin polymerization inhibitor (latrunculin A) treatments significantly inhibited both nuclear deformation and CC dynamics compared to control cells treated with vehicle only (Fig 3B–3D, S12 Fig and S13 Fig). Next, at six days post-differentiation, we counted the number of CC clusters in drug-treated DKO cells and found a significantly larger number of CC clusters in cells treated with blebbistatin and latrunculin A compared to that in the control (Fig 3E). Although actin can affect mechanotransduction and partly nuclear deformation via LINC complexes, these are LamA/C expression-dependent, so it is unlikely that they would influence CC clustering in DKO cells [21,22]. Indeed, no actin cap structure related to mechanotransduction was observed in differentiating DKO cells (S14 Fig). We found that actin cap structure was not observed even in differentiating WT cells. We confirmed that LamA/C were not expressed in WT postmitotic neurons, suggesting that absence of this structure in DKO and WT cells may be related to absence of LamA/C.

Next, we explored how actin participates in nuclear deformation in DKO cells by live imaging. We observed that actin fibers seemed to push and/or pull nucleus during nuclear movement and rotation (and associated nuclear deformation) (S5 Movie and S6 Movie). These results suggest that nuclear deformation driven by actomyosin plays an important role in CC clustering and regulates nuclear architecture reorganization in DKO cells.

### *Ex vivo* nuclear deformation in mouse rod photoreceptor cells

**Fig 4:**
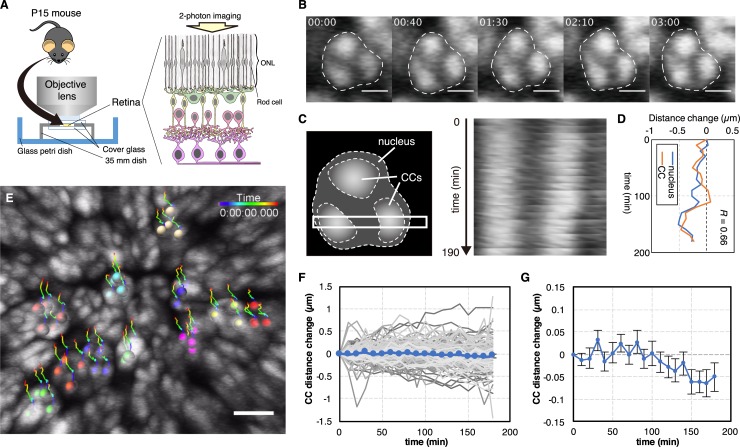
*Ex vivo* live imaging of chromocenter (CC) clusters in mouse rod cells 15 days after birth (P15). (**A**) Live imaging system (see the Supplemental Experimental Procedures for details). ONL: outer nuclear layer. (**B**) Time-lapse single plane images of a single rod-cell nucleus in a P15 mouse. The white dashed lines represent the nuclear edges. Scale bars: 2 μm. (**C**) Schematic cell nucleus with the ROI (white frame, left panel) used to construct the kymograph (right panel). (**D**) Correlated change in the nucleus width and distance between CC clusters. Data are derived from the kymograph in panel (C). (**E**) A representative projected-fluorescence image of the rod-cell nuclei from a P15 mouse (S7 Movie). Center of mass in CC clusters of cells are represented by colored balls. Color-coded lines represent trajectories of CC clusters. Scale bar: 5 μm. (**F, G**) Dynamics of CC distances within individual nuclei (gray lines) and averaged CC distances (blue lines). Error bars represent SEM. *N* = 142 from 20 cells.

Although our experimental observations were made only in model cultured cells, nuclear deformation is generally observed in several systems, including in the mouse retina at birth (post-natal day P0) [23]. However, although the expression of LBR disappears 15 days after birth (P15) in mouse rod cells [2], the dynamic migration of CCs at this developmental stage remains unknown. Therefore, to examine a potential involvement of dynamic nuclear deformation and CC dynamics in mouse retina rod cells at P15, we performed *ex vivo* two-photon live microscopy imaging of the outer nuclear layer of the mouse retina at P15 (Fig 4A).

First, we set a region of interest (ROI) to cross a nucleus and two CCs of photoreceptor cells, and measured the distances between the center of mass of CCs and between the edges of the nucleus within the ROI. We observed a correlation between nuclear deformation and CC migration (the average correlation coefficient was 0.74 ± 0.169 (n = 20); Fig 4B–4D and S7 Movie). Furthermore, the average separation of CC clusters decreased at a rate of ~ 0.015 μm/h (Fig 4E–4G). At this rate, it would take more than 10 days for a CC to travel the distance of an average nucleus diameter (5 μm), consistent with the time scale of complete CC clustering after the loss of LBR expression observed in mouse rod cells [2]. These data support the conclusion of our model cell system and mathematical models that nuclear deformation drives dynamic CC migration and promotes CC clustering in mouse rod photoreceptor cells.

## Discussion

With the advent of Hi-C technology in recent years, it has become clear that cell type-specific nuclear architecture is partially determined by DNA loops, topologically associating domains and compartments [3,4,24]. The whole genome could be split into two spatial compartments, called “A” and “B”, where regions in A compartment tend to interact preferentially with A compartment-associated regions, rather than with the B compartment-associated ones, and *vice versa*. A and B compartment-associated regions correlate with either localization of active chromatin marks and inactive chromatin marks including heterochromatin, respectively [24]. Specifically, B compartments have been shown to be well associated with lamin-associated domains (LADs) and developmentally regulated [25]. Therefore, in addition to genomic interaction, anchoring of heterochromatin to the nuclear envelope is considered to affect the nuclear architecture [5]. Thus, changes in these factors are thought to be involved in the induction of nuclear architecture reorganization during cell differentiation. Recently, it has been shown that the different degrees of affinity between chromatin subdomains could induce genomic reorganization from conventional to inverted nuclear architecture, using polymer block models [26,27]. However, the underlying mechanism driving the long-distance migration of sub-nuclear domains with a fast time scale that cannot be achieved by diffusion alone have thus far remained elusive.

In this study, using the combination of a mathematical model and *in cellulo* and *ex vivo* live imaging, we found that dynamic nuclear deformation is a driving force for the long-range migration of sub-nuclear domains over a short time scale and has a critical effect on nuclear architecture reorganization. Our mathematical model suggested that the inverted nuclear architecture, a representative example of the dramatic nuclear architecture reorganization observed in the developing mouse retina, could be induced by only the dynamic nuclear deformation under the following two assumptions: absence of affinity between the nuclear envelope and CCs, and the CCs only fuse when they are in contact with each other. This implies that the clustering of CCs is a simple matching event that occurs when CCs become free to move and can migrate over a long-range because of nuclear deformation. Furthermore, even when incorporating affinity between the nuclear envelope and CCs, in our mathematical model, the number of fusions between closely located CCs increased in the nucleus with dynamic deformation compared to those without deformation (S8 Movie). This suggests that the dynamic nuclear deformation potentially promotes nuclear architecture reorganization during differentiation, even in many cell systems in which the level of nuclear architecture reorganization is less drastic than that observed for the developing mouse retina.

Our *in cellulo* experiment showed that cytoskeletal forces via actomyosin contractility have an effect on nuclear architecture reorganization. The cytoskeleton including actin fibers and microtubules is bound to the nuclear membrane through the LINC complexes. In addition, the intranuclear part of the LINC complexes associate with the lamins. Cells are considered to transmit mechanical forces from the external environment through the cytoskeleton to the nucleus and to induce gene expression programs to respond to them [28]. However, this mechanotransduction is reported to be LamA/C expression-dependent [21,22]. Therefore, at least in our DKO cells, the suppression of CC clustering can be induced by nuclear deformation impairment induced by actomyosin inhibition, but not by changes in gene expression program stemming from impairment of mechanotransduction through the LINC complexes.

On the other hand, the stiffness and shape of the nucleus are also controlled by the lamin composition in the nuclear membrane and the degree of chromatin condensation [29,30]. Thus, it is possible that treatment with actomyosin inhibitors in DKO cells altered genome-wide gene expression and chromatin organization via unknown mechanisms, thereby affecting nuclear deformation. A small fraction of filamentous actin is present in nucleus and have been suggested to be involved in various nuclear function including transcriptional regulation [31,32]. A recent study reported that a cell-cycle-specific and spatiotemporally controlled form of nuclear actin filaments reorganizes the mammalian nucleus after mitosis [33]. Thus, nuclear actin may be involved in dynamic nuclear deformation and nuclear architecture reorganization. Future research is expected to shed light on these issues.

The nuclear deformability is thought to be related to cell migration [34] and differs greatly depending on the cell type. If the cells migrate within the dense tissues, they need to go through a narrow space. At that time, the cell nucleus, which is the largest organelle in the cell, can be a major obstacle. However, the nuclei of cells moving in dense tissues, including invading cancer cells, are relatively deformable, promoting cell migration [35]. Interestingly, in the neuroepithelium of the central nervous system, including the retinal tissue in developing vertebrates, a phenomenon called interkinetic nuclear migration (IKNM) accompanied by nuclear migration is frequently observed [36]. Neuroepithelium is a sheet of polarized cells that extend from an apical surface to a basal lamina. Mitotic events in a proliferating neuroepithelium are restricted to the apical pole. Mitosis at the apical surface is followed by a basal descent of the nucleus, and an ascent back to the apical surface for the next mitosis [36]. During the retina formation process in zebrafish embryos, actomyosin-dependent IKNM accompanied with obvious nuclear deformation has been recognized by live imaging [37]. IKNM accompanied by nuclear deformation is also recognized in *ex vivo* imaging in mouse P0 stage [23]. It is possible that IKNM may occur even after the P15 stage when LamA/C and LBR are not expressed. Although IKNM could not be confirmed during imaging period in our *ex vivo* imaging, considerable nuclear deformation within the retinal tissue at P15 mouse was observed. Thus, it is possible that nuclear deformation due to IKNM or other factors may promote genomic reorganization during developmental process. On the other hand, in *lama* and *lbr* DKO mice, CC clustering is observed not only within the central nervous system but also in other tissues, suggesting that this might not be a central nervous system specific phenomenon. The developmental process in mammals takes tens of days, accompanied by cellular migration between dense tissues and by external forces due to tissue-level deformation. Therefore, there is a possibility that the cellular migration ability may be adjusted by appropriately controlling the nuclear deformability. It is also possible that such nuclear deformation may promote chromatin movement and genomic reorganization. However, our *in cellulo* model system and *in vivo* tissues differ in aspects such as the surrounding extracellular matrix and the environmental complexity. Therefore, whether nuclear deformation induces genomic reorganization within *in vivo* tissues is yet to be elucidated.

In addition to studies on mouse rod cells, nuclear architecture reorganization during differentiation has also been observed in other systems, including *HoxB* cluster relocalization, “chromosome kissing” during X chromosome inactivation in differentiating mESCs [38,39], choice of a single olfactory receptor gene for expression [7,40] and relocalization of the immunoglobulin heavy-chain gene cluster in differentiating pre-B cells [41,42]. Although these are outstanding examples at specific genomic loci, similar genomic reorganization is thought to occur on a genome-wide level during the cell differentiation process. In this study, our results revealed that dynamic nuclear deformation performs an important function in nuclear architecture reorganization. These findings indicate that the common phenomenon of dynamic nuclear deformation, which accompanies dynamic cell behavior [43–46], could be an underlying driving force of nuclear architecture reorganization.

## Materials and methods

### Experimental model and subject details

#### mESC culture

WT mESCs (Bruce 4 C57BL/6J, male, EMD Millipore, Billerica, MA) and later derivatives were cultured under 2i conditions (StemSure D-MEM [Wako Pure Chemicals, Osaka, Japan], 15% of fetal bovine serum, 0.1 mM β-mercaptoethanol, 1 × MEM nonessential amino acids [Wako Pure Chemicals], a 2 mM L-alanyl-L-glutamine solution [Wako Pure Chemicals], 1000 U/mL LIF [Wako Pure Chemicals], 20 mg/mL gentamicin [Wako Pure Chemicals], 3 mM CHIR99021, and 1 mM PD0325901) in a 0.1% gelatin-coated dish.

#### *Lbr/LamA* DKO cell lines

To disrupt *Lama* and *Lbr* genes simultaneously, we applied a CRISPR-Cas9 system [15,16]. In particular, we used Cas9 nickase that is reported to have fewer off-target effects [17]. To increase the gene disruption rate and simplify detection of the deletion mutation, we designed two sets of sgRNAs (S1A Fig). Next, 5 × 10^4^ WT mESCs were seeded in a gelatin-coated 24-well plate and cultured for 12 h. The cells were transfected with 90 ng each of pKLV-EF-LMNA-1L, -1R, -2L, -2R, and pKLV-EF-LBR-1L, -1R, -2L and -2R and 300 ng of pBSKDB-CBh-Cas9n-pA using Lipofectamine 2000 (Life Technologies, Gaithersburg, MD). After 4 h, the medium containing Lipofectamine was replaced with a fresh regular medium. At 12 h post-transfection, the cells were treated with puromycin (2 μg/mL) for 36 h. Then, the medium was replaced with a fresh medium without puromycin and stood incubating for 48 h. After that, the cells were trypsinized and seeded in a gelatin-coated 10-cm culture dish. Seven days later, we picked 48 colonies and briefly performed PCR to detect gene disruption using specific primers (Lmna-F-gPCR and Lmna-F-gPCR for *Lmna*; Lbr-F-gPCR and Lbr-R-gPC for *Lbr*, S2 Table). Next, some of the candidate clones were subjected to immunofluorescence assays using anti-LBR, anti-LamA/C, and anti-lamin B1 antibodies (Fig 1B and S1B Fig). We obtained four clones (DKO-4, -33, -36, and -48) without LamA/C and LBR expression (S1B Fig). To examine the nucleotide sequence changes in these cells, we first performed southern blots using *Lmna*, *Lbr*, and *Amp* probes as previously described [47]. Probes for *Lmna* and *Lbr* were prepared by means of universal M13 primers, the PCR DIG Probe Synthesis Kit (Roche Diagnostics, Mannheim, Germany) and pBSK-Lmna-probe and pBSK-Lbr-probe, respectively (S2 Table). For *LamA* and *Amp* detection, genomic DNA was digested with SpeI. For *Lbr* detection, genomic DNA was digested with BmtI and BstEII (S1C Fig). Furthermore, we determined genomic DNA sequence around CRISPR target sites of *LamA* and *Lbr* in clones DKO-4 and -36, in which both genes were expected to be inactivated (S1D and S1E Fig). In this study, we mainly used clone DKO-4 as representative of the DKO cell lines unless otherwise indicated. We confirmed that the number of CC clusters steadily decreases during differentiation process in DKO-36 cells. The average number of CC clusters in DKO-36 mESCs, neural stem cells (NSCs) and neurons at 16 days post-differentiation were 20.3 ± 6.69, 7.96 ± 2.87 and 4.49 ± 3.00, respectively. DKO-4 mESCs expressed pluripotency markers, Nanog and Oct4 (S2 Fig), but not NSC and neuron markers (S3 Fig and S4 Fig).

#### Differentiation into postmitotic neurons

NSC differentiation was induced using a protocol adapted from Conti et al. [48]. Briefly, mESCs were trypsinized and resuspended in the N2B27 medium [1:1 mix of DMEM/F12 (Thermo Fisher Scientific) supplemented with N2 (Life Technologies) and Neurobasal medium (Thermo Fisher Scientific) supplemented with B27 (Life Technologies)]. Next, 2 × 10^5^ cells were seeded in gelatin-coated 6-cm plates. At 5 days post-differentiation, the cells were resuspended in the N2 expansion medium [49] and plated in uncoated T-75 flasks to allow for neurosphere outgrowth. Cell aggregates were allowed to settle under the force of gravity in a 15-mL tube for 10 min. The cells were seeded in 6 mL of a fresh NS expansion medium in poly-ornithine/laminin-coated 6-cm plates. Following outgrowth of cells (3–7 days), the entire population was trypsinized and seeded as single cells in poly-ornithine/laminin-coated 6-cm plates in the expansion medium. After several passages, the cultures were uniformly positive for an NSC marker, Nestin (S2 Fig to S5 Fig).

For neuronal differentiation, NSCs were trypsinized, and 5 × 10^3^ cells were seeded in each well of a poly-ornithine/laminin-coated 8μ-slide (ibidi, Martinsried, Germany) in the N2B27 medium supplemented with FGF-2 (5 ng/mL) (neural induction medium). Half of the medium was replaced every 2–3 days to maintain conditioning of the medium. After the cells spent 6 days under these conditions, we replaced the media with a 50:50 mixture of DMEM/F12 and Neurobasal medium (Thermo Fisher Scientific, Waltham, MA) supplemented with 0.25× N2 plus 1× B27 and without EGF or FGF. At 9 days postdifferentiation, the cultures were positive for a neuron marker, βTub III (S2 Fig to S5 Fig).

#### Mice

The use of animals in the present study was in accordance with the Guidelines for Animal Experiments of Nagoya University. All animal experiments were conducted with the approval of the Animal Research Committee in Nagoya University. C57BL/6J mice were purchased from CLEA (Shizuoka, Japan), and maintained under a cycle of 12-hour light and 12-hour dark with free access to food and water. For *ex vivo* imaging, 15-days-old C57BL/6J mice were used.

### Mathematical model details

#### Model development

We extended the model from a previous study [12]. The key point of our modeling is to describe the dynamics of subnuclear compartments by linking them to domains using phase-field functions (Fig 2A). We first designed each domain by means of energy functions defined by the nucleus, *ϕ*_0_(**x**,*t*), chromosome territories, *ϕ*_*m*_(**x**,*t*) (1≤*m*≤*N*), heterochromatin, *ψ*(**x**,*t*) and chromocenters (CCs), *ψ*_0_(**x**,*t*), where **x**∈*Ω* in **R**^*n*^ and *t*>0. *N* represents the total number of chromosomes in the nucleus, and we typically chose *N* = 8 for representative simulations.

We first defined the basal free energy functions using Ginzburg-Landau free energy [50,51] for the nucleus, chromosome territories, heterochromatin and CCs according to the following equation:

$$\begin{array}{r} E_{0}=\int_{\Omega }\left[\frac{\epsilon_{0}^{2}}{2}|\nabla \phi_{0}{|}^{2}+g(\phi_{0})\right]dx+\sum_{m=1}^{N}\int_{\Omega }\left[\frac{\epsilon_{\phi }^{2}}{2}|\nabla \phi_{m}{|}^{2}+g(\phi_{m})\right]dx+\int_{\Omega }\left[\frac{\epsilon_{\psi }^{2}}{2}|\nabla \psi {|}^{2}+g(\psi )\right]dx\\ +\int_{\Omega }\left[\frac{\epsilon_{\psi_{0}}^{2}}{2}|\nabla \psi_{0}{|}^{2}+g(\psi_{0})\right]dx \end{array}$$

where $\epsilon_{0},\epsilon_{\phi },\epsilon_{\psi },\epsilon_{\psi_{0}}>0$ are gradient coefficients.

Second, we formed subnuclear compartments using a restriction condition and by defining volumes. The restriction conditions are given as follows:

**(*S***_**1**_**)** each chromosome should be restricted in the nuclear domain [52],

**(*S***_**2**_**)** no heterochromatin domains should escape from the given chromosome domain [52],

**(*S***_**3**_**)** the chromosome domains tend to be separated from each other and constitute separate territories [19],

**(*S***_**4**_**)** no CC should escape from the given heterochromatin domain and chromosome domain [52].

The conditions, *S*_1_–*S*_4_ are described as

$$\begin{array}{r} \underset{S1}{\underset{︸}{E_{1}=\beta_{0}\sum_{m=1}^{N}\int_{\Omega }h\left(\phi_{0}\right)h\left(\phi_{m}\right)dx}}+\underset{S2}{\underset{︸}{\beta_{\psi }\int_{\Omega }\left[1-\sum_{m=1}^{N}h\left(\phi_{m}\right)\right]h\left(\psi \right)dx}}+\underset{S3}{\underset{︸}{\beta_{\phi }\sum_{m\neq n}^{N}\int_{\Omega }h\left(\phi_{n}\right)h\left(\phi_{m}\right)dx}}\\ +\underset{S4}{\underset{︸}{\beta_{\psi_{0}}\left\{\int_{\Omega }\left[1-h\left(\psi \right)\right]h\left(\psi_{0}\right)dx+\int_{\Omega }\left[1-\sum_{m=1}^{N}h\left(\phi_{m}\right)\right]h\left(\psi_{0}\right)dx\right\}}}, \end{array}$$

where *β*_0_, *β*_*ψ*_, *β*_*ϕ*_ and $\beta_{\psi_{0}}$ are positive constants and indicate the intensity of domain territories. *h*(*ϕ*_*m*_) is given for $h(\phi_{m})=\phi_{m}^{3}(10-15\phi_{m}+6\phi_{m}^{2})$, which is technically used to induce energetic asymmetry between *ϕ* = 0 and *ϕ* = 1 to drive the interface while keeping *ϕ* = 0 and *ϕ* = 1 local minima. *E*_0_ and *E*_1_ are fundamental formulations for setting up nuclear and subnuclear compartments.

Next, we define chromosome and heterochromatin volumes according to the following equation:

$$\begin{array}{r} E_{2}=\alpha_{0}{\left[V_{0}(t)-\sum_{m=1}^{N}V_{m}(t)\right]}^{2}+\alpha_{V}\sum_{m=1}^{N}[V_{m}(t)-{\bar{V}}_{m}(t){]}^{2}+\alpha_{v}\sum_{m=1}^{N}[v_{m}(t)-{\bar{v}}_{m}(t){]}^{2}+\alpha_{v_{0}}\sum_{m=1}^{N}[v_{m_{0}}(t)-{\bar{v}}_{m_{0}}(t){]}^{2} \end{array}$$

where

$$V_{0}(t)=\int_{\Omega }[1-h(\phi_{0})]dx,$$

$$\begin{array}{r} V_{m}(t)=\int_{\Omega }h(\phi_{m})dx,\;v_{m}(t)=\int_{\Omega }h(\phi_{m})h(\psi )dx,\;v_{m_{0}}(t)=\int_{\Omega }h(\phi_{m})h(\psi_{0})dx. \end{array}$$

*V*_0_(*t*) is the volume of nucleus and ${\bar{V}}_{m}(t)$, ${\bar{v}}_{m}(t)$ and ${\bar{v}}_{m_{0}}(t)$ are the target volumes of chromosomes, heterochromatin, and CCs, respectively, which is determined and calculated by the size of cell nucleus at time *t* divided by each number. That is, the target volumes are temporally changed depending on the nuclear volume changed temporally. We define ${\bar{V}}_{m}(t)=V_{0}(t)/N,{\bar{v}}_{m}(t)=0.5\times {\bar{V}}_{m}(t)$ and ${\bar{v}}_{m_{0}}(t)=0.1\times {\bar{V}}_{m}(t)$. In the case of fixed nucleus with a constant volume *V*_0_, we set ${\bar{V}}_{m}(t)=\frac{V_{0}}{N}(\equiv {\tilde{V}}_{m}),{\bar{v}}_{m}(t)=0.5\times {\tilde{V}}_{m}(\equiv {\tilde{v}}_{m})$, ${\bar{v}}_{m_{0}}(t)=0.1\times {\tilde{V}}_{m}(\equiv {\tilde{v}}_{m_{0}})$. The first term of *E*_2_ ensures the condition that the nucleus is fully occupied by chromosomes.

As an additional condition that is unnecessary to regenerate inverted nuclear architecture but is required to produce conventional nuclear architecture, we can describe the role of LBR and/or LamA/C between heterochromatin and the nuclear envelope [2] in the model by the affinity between nuclear function, *ϕ*_0_, and heterochromatin domain function, *ψ*, as follows:

$$\begin{array}{r} E_{3}=\gamma \int_{\Omega }\nabla h(\phi_{0})\cdot \nabla h(\psi )dx, \end{array}$$

where *γ* is a constant that determines the strength of the affinity. Note that *γ* > 0 reflects the presence of LBR and/or Lam A/C on the interior of the nuclear envelope and their tethering of heterochromatin to the nuclear periphery. In contrast, *γ* = 0 represents the absence of LBR and Lam A/C expression and means that heterochromatin and the nuclear envelope do not interact with detectable affinity.

Now, the total energy of subnuclear compartments is expressed as:

$$E=E_{0}+E_{1}+E_{2}+E_{3}.$$ (1)

Then, we take the functional derivatives of Eq (1) with respect to *ϕ*_*m*_ (1≤*m*≤*N*), *ψ* and *ψ*_0_, which drives the system to evolve with time, satisfying:

$$\begin{array}{r} \frac{\partial \phi }{\partial t}=-\mu \frac{\delta E}{\delta \phi }\;(1\leq m\leq N),\;\frac{\partial \psi }{\partial t}=-\mu \frac{\delta E}{\delta \psi },\;\frac{\partial \psi_{0}}{\partial t}=-\mu \frac{\delta E}{\delta \psi_{0}} \end{array}$$

where *μ* is a positive constant that represents the mobility of each phase-field. Calculating the above equations yields the reaction-diffusion model as follows:

$$\begin{array}{l} \mu^{-1}\frac{\partial \phi_{m}}{\partial t}=\epsilon_{\phi }^{2}\nabla^{2}\phi_{m}+\phi_{m}(1-\phi_{m})\left[\phi_{m}-\frac{1}{2}-A_{m}\phi_{m}(1-\phi_{m})\right],(1\leq m\leq N)\\ \mu^{-1}\frac{\partial \psi }{\partial t}=\epsilon_{\psi }^{2}\nabla^{2}\psi +\psi (1-\psi )\left[\psi -\frac{1}{2}-B\psi (1-\psi )\right]\\ \mu^{-1}\frac{\partial \psi_{0}}{\partial t}=\epsilon_{\psi_{0}}^{2}\nabla^{2}\psi_{0}+\psi_{0}(1-\psi_{0})\left[\psi_{0}-\frac{1}{2}-B_{0}\psi_{0}(1-\psi_{0})\right] \end{array}$$ (2)

where

$$\begin{array}{l} A_{m}=-60\alpha_{0}\left[\int_{\Omega }[1-h(\phi_{0})]dx-\sum_{m=1}^{N}V_{m}\right]+60\alpha_{V}(V_{m}-{\bar{V}}_{m})\\ \;+60\alpha_{v}(v_{m}-{\bar{v}}_{m})h(\psi )+60\alpha_{v_{0}}(v_{m_{0}}-{\bar{v}}_{m_{0}})h(\psi_{0})\\ \;+30\beta_{0}h(\phi_{0})+30\beta_{\phi }[\chi -h(\phi_{m})]-30\beta_{\psi }h(\psi )-30\beta_{\psi_{0}}h(\psi_{0}),\\ B=60\alpha_{v}\sum_{m=1}^{N}\left[\left(v_{m}-{\bar{v}}_{m})h(\phi_{m}\right)\right]+30\beta_{\psi }(1-\chi )-30\beta_{\psi_{0}}h(\psi_{0})-30\gamma \nabla^{2}h(\phi_{0}),\\ B_{0}=60\alpha_{v}\sum_{m=1}^{N}\left[\left(v_{m_{0}}-{\bar{v}}_{m_{0}})h(\phi_{m}\right)\right]+30\beta_{\psi_{0}}(1-\chi )+30\beta_{\psi_{0}}(1-h(\psi_{0})),\\ \chi =\sum_{m=1}^{N}h(\phi_{m}). \end{array}$$

Note that to generate conventional nuclear architecture as the initial conditions, we solved the system (2) with >0, but *γ* = 0 is always assumed in the simulations for inverted nuclear architecture because we do not need the condition for affinity between heterochromatin and the nuclear envelope in the nuclear architecture reorganization process.

Now, we nondimensionalize the model (2) for time scale *T* and spatial scale *L* incorporating $t=T\tilde{t}$ and $x=L\tilde{x}$ into the model (2). Let us define the mobility constant, *μ*, as:

$$\mu =T^{-1}$$

and set:

$$\begin{array}{r} \varepsilon_{\phi }^{2}=\epsilon_{\phi }^{2}L^{-2},\;\varepsilon_{\psi }^{2}=\epsilon_{\psi }^{2}L^{-2}. \end{array}$$

We then obtain the following system by removing the tilde:

$$\begin{array}{l} \frac{\partial \phi_{m}}{\partial t}=\varepsilon_{\phi }^{2}\nabla^{2}\phi_{m}+\phi_{m}(1-\phi_{m})\left[\phi_{m}-\frac{1}{2}-A_{m}\phi_{m}(1-\phi_{m})\right],\;(1\leq m\leq N)\\ \frac{\partial \psi }{\partial t}=\varepsilon_{\psi }^{2}\nabla^{2}\psi +\psi (1-\psi )\left[\psi -\frac{1}{2}-B\psi (1-\psi )\right],\\ \frac{\partial \psi_{0}}{\partial t}=\varepsilon_{\psi_{0}}^{2}\nabla^{2}\psi_{0}+\psi_{0}(1-\psi_{0})\left[\psi_{0}-\frac{1}{2}-B_{0}\psi_{0}(1-\psi_{0})\right]. \end{array}$$ (3)

In the model, we assumed that the nucleus changes its size and shape independently of chromatin states, so that the phase-field *ϕ*_0_ is given to be an independent function, describing the nucleus unrelated to the other phase-field functions. That is, we directly control the nuclear deformation using the following equation:

$$\mu^{-1}\frac{\partial \phi_{0}}{\partial t}=\epsilon_{0}^{2}\nabla^{2}\phi_{0}+\phi_{0}(1-\phi_{0})\left[\phi_{0}-\frac{1}{2}-D(x,t)\phi_{0}(1-\phi_{0})\right],$$

where D(x,t) is a deformation function, which controls movement of the interface of the nuclear phase-field. For example, to make a circular nucleus from an elliptical one in 2D that is initially given, we define it as

$$D(x,t)=\left\{ \begin{array}{ll} A_{0}dist(x,r(t)) & if\;dist(x,r(t))\leq 0\\ -A_{0}dist(x,r(t)) & if\;dist(x,r(t))>0 \end{array}\right.$$ (4)

where **x**_**0**_ is the center coordinate of the nucleus, *r*(*t*) is the radius of the circle, and *dist*(**x**,*r*) = |**x**−**x**_**0**_|−*r*(*t*). *A*_0_ is the positive value controlling the speed of deformation, so that we can make the difference of dynamic degree in deformation by adjusting *A*_0_. By defining *r*(*t*) as a decreasing function, we can generate the decrease in nuclear size.

#### Fusion of heterochromatin regions and CCs

In the above modeling, we implicitly assumed the fact that heterochromatin regions and CCs in different chromosomes are fused when they are in contact with each other [6,9,10]. We described all heterochromatin domains and CCs in a nucleus using a single phase-field function. Such description leads to the fusion when the interfaces of two separate domains are close, as shown in S9B and S9C Fig.

#### Parameter validation

Because we captured subnuclear compartments as domains, the key element determining the dynamics of heterochromatin or CC clustering is the interface of phase-field function. The interface region, {**x**|0<*ϕ*(**x**)<1}, can be interpreted as a confinement region of chromatin movement as shown in S9D Fig. That is, the thickness of the interface, denoted by *γ*, can be interpreted as the radius of the confinement region, which was found to have a value less than 4 μm [11].

In fact, we can explicitly calculate the thickness of the interface [53] as follows:

$$\delta =4\sqrt{2}\epsilon_{\phi }{tanh}^{-1}(1-2\lambda )$$

where *λ* is a given constant defining the interface region, such that *λ*≤*ϕ*≤1−*λ*. Although it is very difficult to estimate all parameters of the model from experimental data, we validated our representative parameters by using the scale of the confinement region (*δ*) and the spatial scale of nuclear size.

For the simulations, *Ω* is given by *L*_*x*_×*L*_*y*_, where *L*_*x*_ and *L*_*y*_ are horizontal and perpendicular lengths, respectively, in the square shown in S9A Fig. We estimated the spatial scale directly from the images of our experiments and from a previous report [6], where the size of the nucleus in two dimensions is approximated to 4 μm to 8 μm for the short diameter (the *y*-axis diameter), $L_{sd}^{d}$, and 6 μm to 12 μm for the long diameter (the *x* -axis diameter), $L_{ld}^{d}$. We simulated the nondimensionalized system for *L*_*x*_×*L*_*y*_ = 1.8×1.8 square nucleus and *L*_*ld*_×*L*_*sd*_ = 1.6×1.0 elliptic nucleus by the scaling by means of $L_{sd}^{d}=6.25\;\mu m$. That is, the dimensional length of the short diameter and long diameter of the elliptic nucleus are expressed as $L_{ld}^{d}\times L_{sd}^{d}=9\;\mu m\times 6.25\;\mu m$. Therefore, the nuclear size is chosen in the feasible range with respect to the real-world data. We also chose *ε*_*ϕ*_ such that *δ* is in the feasible range. The detailed values of *ε*_*ϕ*_ in S3 Table satisfy $\epsilon_{\phi }^{2}=\varepsilon_{\phi }^{2}L^{2}(\mu m^{2})$ and

$$\delta =4\sqrt{2}\epsilon_{\phi }{tanh}^{-1}(1-2\lambda )\in (0.275\;\mu m,0.390\;\mu m)$$

for *λ* = 0.15.

On the other hand, we estimated time scale *T* using the time scale of nuclear deformation. To validate the time scale that we chose for the simulation, we compared qualitative dynamics and temporal data that were obtained from a previous report [6] and in our experiments of model cell system. Note that the scale of interface thickness (*i*.*e*., the confinement length scale of subnuclear compartment movement) is independent from the time scale of the model dynamics because we defined the mobility of phase-fields as *μ* = *T*^−1^ in system. That is, the time scale of nuclear architecture reorganization process is determined by the time scale of nuclear deformation, *T*. We also ran simulations with a nondimensionalized system (3) so that the results are consistent without dependence on the specific nuclear size. The detailed representative parameters are given in S3 Table.

The main results in the paper was presented for *N* = 8, but the effect of dynamic deformation on CC clustering is not dependent on the number of *N*. The example result for the case of *N* = 12 is shown in S9E Fig.

#### The measure of nuclear deformation

In numerical simulations, we obtained dynamic deformation of the nucleus, with fixing of the center of the nucleus, and we created several shapes randomly. That is, the deformation magnitude of all shapes can be quantified via measurement how much the boundary of a nucleus differs from the boundary of a circle of the same area (Fig 2H). We calculated the deformation degree by

$$M_{df}(\%)=\frac{Total\;area\;of\;deviated\;from\;circle}{Area\;of\;nucleus}\times 100.$$

The case of low deformation degree (Fig 2G) was formulated with choosing smaller value of *A*_0_ that chosen in the case of high deformation degree (Fig 2E). The average of total deformation degree ($\int_{0}^{24h}M_{df}(t)dt)/24h$) of Fig 2G was about 30% smaller than that of Fig 2E.

#### The effect of nuclear size on nuclear architecture reorganization

During the nuclear architecture reorganization in a rod photoreceptor cell, we found that this process is accompanied by a 40% reduction in nuclear volume (by 20% of the area in 2D) and convergence of its shape from an ellipsoidal to spherical one during the differentiation [6]. To test whether the effect of the nuclear size decrease critically affects the clustering of CCs, we conducted simulations with a 20% decrease in the nuclear area. In many simulations, we observed that the nuclear size decrease does not have such a critical effect on clustering of CCs (S10 Fig). We could not find that the decrease in nuclear size promotes CC clustering advantageously without sufficient deformation of the nucleus, indicating that the distance between CCs can be decreased because of a decrease in the nucleus size but its effect is negligible, and the dynamic nuclear deformation is a sufficient condition for the nuclear architecture reorganization.

#### Nuclear deformation definition

The nucleus of mouse rod photoreceptor cell has been described to be deformed from an ellipsoidal to spherical shape over time scales of several weeks during differentiation. Here, we refer this deformation as “quasi-static” nuclear deformation. We defined the time scale of nuclear deformation, *T*_*d*_, by the time for moves of the nuclear envelop 1 μm. Then, the speed of nuclear deformation (*S*_*d*_) can be estimated as $S_{d}=\frac{1}{T_{d}}(\mu mh^{-1})$. We also defined the limited time for the nuclear architecture reorganization as *T*_*p*_, which is reported to be from several dozen days to several months in a rod photoreceptor cell [6], and we set *T*_*p*_ = 41 days in the representative parameter set. On the other hand, it was found that specific genomic regions and subnuclear compartments are confined and immobile over distances greater than 0.4 μm for more than 1 hour scale [11]. That is, the movement speed of a subnuclear compartment (*S*_*c*_) can be estimated as *S*_*c*_≤0.4 (*μmh*^−1^).

Now, by comparing the time scale or speed of nuclear deformation with the speed of the subnuclear compartment, we classified nuclear deformation as follows:

- **Static state nucleus** when *S*_*d*_ = 0,
- **Quasi-static nuclear deformation** when *S*_*d*_≪*S*_*c*_ and *T*_*d*_≈*T*_*p*_,
- **Dynamic nuclear deformation** when *S*_*d*_≫*S*_*c*_ and *T*_*d*_≪*T*_*p*_.

In the simulation, we typically chose *S*_*d*_≈0.088 (*μmh*^−1^) with *T*_*d*_ = *T*_*p*_ days for quasi-static deformation, and *S*_*d*_≈2.4 (*μmh*^−1^) with *T*_*d*_ = 25 min for dynamic deformation.

## Supporting information

S1 Text 1–1. Plasmid construction
1–2. Immunofluorescence assays
1–3. Counting of chromocenters
1–4. Establishment of LBR revertant cells
1–5. Live-cell imaging without drug treatment
1–6. Cytoskeletal drug treatment and live imaging
1–7. Actin fiber imaging
1–8. *Ex vivo* live imaging
1–9. Statistical analysis.
(DOCX)

S1 Movie Live imaging of *Lbr/LamA* double-knockout (DKO) neuronal cells at 3 days postdifferentiation on a longer time scale, example 1. DKO neural stem cells (NSCs) were induced to differentiate into neurons. At 3 days postdifferentiation, the cells were stained with SiR-Hoechst and subjected to live imaging by confocal microscopy. Images were taken at 30-min intervals for 44 h. Z-series of 25 focal planes with a step size of 0.5 μm were acquired. The cells actively migrated during the imaging period. Thus, the nucleus was centered to make it easier to follow. The maximal projected image sequence shows dynamic nuclear deformation and chromocenter (CC) clustering. Scale bars: 10 μm.
(MOV)

S2 Movie Live imaging of *Lbr/LamA* double-knockout (DKO) neuronal cells at 3 days postdifferentiation on a longer time scale, example 2. DKO neural stem cells (NSCs) were induced to differentiate into neurons. At 3 days postdifferentiation, the cells were stained with SiR-Hoechst and subjected to live imaging by confocal microscopy. Images were taken at 30-min intervals for 44 h. Z-series of 25 focal planes with a step size of 0.5 μm were acquired. Cells actively migrated during the imaging period. Thus, the nucleus was centered to make it easier to follow. The maximal projected image sequence shows dynamic nuclear deformation and chromocenter (CC) clustering. Scale bars: 10 μm.
(MOV)

S3 Movie Live imaging of *Lbr/LamA* double-knockout (DKO) neuronal cells at 3 days postdifferentiation on a longer time scale, example 3. DKO neural stem cells (NSCs) were induced to differentiate into neurons. At 3 days postdifferentiation, the cells were stained with SiR-Hoechst and subjected to live imaging by confocal microscopy. Images were taken at 30-min intervals for 44 h. A Z-series of 25 focal planes with a step size of 0.5 μm were acquired. Cells actively migrated during the imaging period. Thus, the nucleus was centered to make it easier to follow. The maximum projected image sequence shows dynamic nuclear deformation and chromocenter (CC) clustering. Scale bars: 10 μm.
(MOV)

S4 Movie Results of numerical simulation of chromocenter (CC) clustering by dynamic nuclear deformation. The period of simulation is approximately 33 h.
(MOV)

S5 Movie Live imaging of actin dynamics in *Lbr/LamA* double-knockout (DKO) neuronal cells at 3 days postdifferentiation, example 1. DKO neural stem cells (NSCs) were induced to differentiate into neurons. At 3 days postdifferentiation, the cells were stained with SiR-Actin and Vybrant DyeCycle Orange Stain and subjected to live imaging by confocal microscopy. Images were taken at 15-min intervals for 3 h. A Z-series of 77 focal planes with a step size of 0.2 μm were acquired. The maximum projected image sequence shows dynamic actin movement. In this movie, three images are connected horizontally. Left, middle, and right side of images represent merged, nucleus (Vybrant DyeCycle Orange Stain), and actins (SiR-Actin), respectively. Scale bars: 10 μm.
(MOV)

S6 Movie Live imaging of actin dynamics in *Lbr/LamA* double-knockout (DKO) neuronal cells at 3 days postdifferentiation, example 2. DKO neural stem cells (NSCs) were induced to differentiate into neurons. At 3 days postdifferentiation, the cells were stained with SiR-Actin and Vybrant DyeCycle Orange Stain and subjected to live imaging by confocal microscopy. Images were taken at 15-min intervals for 3 h. A Z-series of 77 focal planes with a step size of 0.2 μm were acquired. The maximum projected image sequence shows dynamic actin movement. In this movie, three images are connected horizontally. Left, middle, and right side of images represent merged, nucleus (Vybrant DyeCycle Orange Stain), and actins (SiR-Actin), respectively. Scale bars: 10 μm.
(MOV)

S7 Movie *Ex vivo* live imaging of P15 mouse rod cells. Retinal tissue was excised from P15 mouse, stained with Hoechst 33342 and subjected to live imaging using two-photon microscopy. Images were taken at 10-min intervals for 3 h. Z-series of 103 focal planes with a step size of 0.2 μm were acquired. Center of mass in chromocenter (CC) clusters of some cells are represented by colored balls. Color coded lines represent trajectories of CC clusters. Scale bar: 5 μm.
(MOV)

S8 Movie Dynamics deformation with affinity between heterochromatin and nuclear envelop. (MOV)

S1 Fig Establishment of DKO cell lines. **(A)** The strategy for targeted gene inactivation for establishment of the *Lbr*/*Lama* double knockout (DKO) cell lines is shown in the left panel. Grey and black boxes indicate untranslated regions and coding sequences, respectively. The right-hand panel shows schematic representations of target sequences of clustered regularly interspaced short palindromic repeat (CRISPR)-Cas9 nickase (Cas9n) used in this study. The target and protospacer adjacent motif (PAM) sequences are indicated with blue and red letters, respectively. **(B)** Immunofluorescence staining showing expression of LBR, LamA/C, and lamin B1 in wild-type (WT) and DKO cells in the left panel. We established four DKO cell lines. Scale bar: 10 μm. **(C)** Southern blot analyses showing a CRISPR-induced deletion in the DKO cell clones. An asterisk represents a nonspecific band. Some obscure bands are highlighted by arrows. The right-hand panel represents genomic structures of *Lama* and *Lbr*. Blue lines represent the Southern blot probe used in this study. **(D, E)** Sequence alignment of a genomic DNA region around CRISPR target sites of *Lama* and *Lbr* in clones DKO-4 and -36 and WT.
(PDF)

S2 Fig Representative immunofluorescent staining of pluripotency markers, Nanog and Oct4, in WT and DKO-4 embryonic stem cells (ESCs), neural stem cells (NSCs), and neurons at 9 days postdifferentiation. Nuclei were stained with Hoechst 33342. Scale bar, 50 μm.
(EPS)

S3 Fig Representative immunofluorescent staining of a neural stem cell marker, Nestin, in WT and DKO-4 embryonic stem cells (ESCs), neural stem cells (NSCs) and neurons at 9 days postdifferentiation. Nuclei were stained with Hoechst 33342. Scale bar, 50 μm.
(EPS)

S4 Fig Representative immunofluorescence of a neuron marker, βTub III, in WT and DKO-4 embryonic stem cells (ESCs), neural stem cells (NSCs), and neurons at 9 days postdifferentiation. The nuclei were stained with Hoechst 33342. Scale bar, 50 μm.
(EPS)

S5 Fig Representative immunofluorescence of H4K20me3 and H3K4me3 in WT and DKO-4 embryonic stem cells (ESCs), neural stem cells (NSCs), and neurons at 9 days postdifferentiation. Nuclei were stained with Hoechst 33342. We confirmed that localization characteristics of histone marks (H4K20me3 and H3K4me3) are qualitatively similar to those in mouse rod cells [6]. Scale bar, 5 μm.
(EPS)

S6 Fig Correlation between the number of chromocenter clusters and nuclear volume. Nuclear volumes and the numbers of chromocenter clusters in DKO-4 embryonic stem cells (ESCs), neural stem cells (NSCs), and neurons at 6, 9, and 16 days postdifferentiation were measured using Imaris and plotted. The number of chromocenter clusters and nuclear volume correlated (Spearman rho = 0.609, *p* = 5.0298e-58, *n*_ESC_ = 33; *n*_NSC_ = 181; *n*_d6_ = 117; *n*_d9_ = 80; *n*_d16_ = 139).
(EPS)

S7 Fig LBR expression rescues conventional nuclear architecture in postmitotic DKO cells. We established an LBR-reverted DKO-4 ESC cell line (DKO-4+LBR). Then, DKO-4 and DKO-4+LBR ESCs were induced to differentiate into NSCs. The established NSCs were induced to differentiate into neurons. At 6 days postdifferentiation, the cells were fixed with 4% PFA and subjected to immunofluorescence staining using an anti-LBR antibody. Nuclei were stained with Hoechst 33342. Scale bar, 5 μm. LBR-reverted DKO cells did not show inverted nuclear architecture.
(EPS)

S8 Fig Nucleotide sequence of pLR5-CBh-dCas9-mNenoGreen-IRES-Hyg. (PDF)

S9 Fig Model formulation. (**A**) An example of a color plot of subnuclear compartments. The imaging data for a rod cell from a 6-day-old mouse [6] and a simulation example image are shown in the upper and lower panel, respectively. (**B**, **C**) The conditions for heterochromatin fusion. (**D**) The sub-nulcear domain of a subnuclear compartment. The interface thickness of a phase-field function, *δ*, corresponds to the sub-nulcear domain region of the subnuclear compartment. (**E**) Representative simulation for 12 numbers of a chromocenter case.
(TIFF)

S10 Fig The effect of nuclear size. The effect of nuclear size on CC clustering is shown for the same level of a deformation degree. (**A**) The case that nuclear size is fixed. (**B**) The case when nuclear size is decreased by 20%. (**C**) Deformation degrees for panels (A) and (B).
(TIFF)

S11 Fig The number of chromocenter (CC) clusters during differentiation. This data is the same as Fig 1C, but represented as histogram, so that reader can recognize that there is considerable variation in the number of CCs even in cells at 16 days post-differentiation.
(EPS)

S12 Fig Projected nuclear area fluctuations in DKO cells treated with cytoskeletal drugs. A normalized histogram of combined projected nuclear area fluctuations for all the cells and all time points in DKO cells treated with DMSO, blebbistatin, ciliobrevin D, nocodazole, or paclitaxel is shown in the right-hand panel (see the Supplemental Experimental Procedures for details). Solid lines represent Gaussian fitting. Area fluctuations versus time plots for multiple cells are presented in the left panel. *n*_DMSO_ = 1464 from 24 cells; *n*_blebbistatin_ = 1403 from 23 cells; *n*_Latrunculin A_ = 1220 from 20 cells; *n*_ciliobrevin D_ = 488 from eight cells; *n*_nocodazole_ = 427 from seven cells; *n*_paclitaxel_ = 549 from nine cells.
(EPS)

S13 Fig Ensemble- and time-averaged MSD between CC clusters and nuclear center of mass. Ensemble- and time-averaged MSD between CC clusters and nuclear center of mass in cells treated with DMSO, blebbistatin (bleb), nocodazole (noco), paclitaxel (pac), ciliobrevin (cilio) or latrunculin A (LatA) during the 1-h imaging period are shown on a linear scale (*n*_DMSO_ = 194 from 28 cells; *n*_bleb_ = 133 from 21 cells; *n*_cilio_ = 84 from 14 cells; *n*_noco_ = 66 from 12 cells; *n*_pac_ = 158 from 22 cells; *n*_Latrunculin A_ = 141 from 23 cells). All error bars represent the standard error of measurements. Every MSD curve almost reached a plateau in the imaging period, suggesting that diffusible space of CC clusters is a constraint. Using the value of the plateau, we calculated a volume of a spherical confinement of CC movement [54] and found that the volume of spherical confinement in cells treated with blebbistatin and Latrunculin A was approximately three-fold smaller in comparison with the others. (B) Ensemble- and time-averaged MSD between CC clusters and nuclear center of mass in either DKO cells or WT cells treated with DMSO, blebbistatin (bleb), nocodazole (noco) during the 1-h imaging period are shown on a linear scale (nDMSO-DKO = 194 from 28 DKO cells; nbleb-DKO = 133 from 21 DKO cells; nnoco-DKO = 66 from 12 cells; nDMSO-WT = 221 from 18 WT cells; nbleb-WT = 216 from 17 WT cells; nnoco-WT = 114 from 10 WT cells). All error bars represent the standard error of measurements.
(EPS)

S14 Fig Perinuclear actin cap structure was not observed in differentiating DKO cells. (A) and WT cells (B). After 9 days post-differentiation of DKO and WT NSCs, cells were fixed and stained with Alexa 488 phalloidin and Hoechst 33342. In these cells, perinuclear actin cap structure was not observed. (C) Immunofluorescence staining of LBR, LamA/C, and lamin B1 in WT and DKO neurons at 9 days postdifferentiation. LamA/C at the nuclear periphery was not detected in WT cells. Scale bar: 10 μm.
(EPS)

S1 Table A list of the sequences of the oligonucleotides for construction of sgRNA expression vectors used in this study. (XLSX)

S2 Table A list of the nucleotide sequences of primers used in this study. (XLSX)

S3 TableDetails of simulation parameters.The details of representative dimensional/nondimensional parameters are given in the following Table, except for *α*_0_, *α*_*V*_, *α*_*v*_, $\alpha_{v_{0}}$, *β*_0_, *β*_*φ*_, *β*_*ψ*_, $\beta_{\psi_{0}}$, which are the same in both nondimensional and dimensional systems, such that *α*_0_ = 25/6, *α*_*V*_ = 10/6, $\alpha_{v}=\alpha_{v_{0}}=40/3$, *β*_0_ = 5/3, *β*_*ψ*_ = 1, and $\beta_{\psi }=\beta_{\psi_{0}}=1/6$.(DOCX)
