# Supplementary material for: Role of dynamic nuclear deformation on genomic architecture reorganization
Source: PLoS Comput Biol. 2019 Sep 11;15(9):e1007289. doi: 10.1371/journal.pcbi.1007289 (PMC6738595; doi:10.1371/journal.pcbi.1007289)
Supplement: S1 Text — 1–1. Plasmid construction 1–2. Immunofluorescence assays 1–3. Counting of chromocenters 1–4. Establishment of LBR revertant cells 1–5. Live-cell imaging without drug treatment 1–6. Cytoskeletal drug treatment and live imaging 1–7. Actin fiber imaging 1–8. Ex vivo live imaging 1–9. Statistical analysis. (DOCX) [file pcbi.1007289.s001.docx]

**Supporting Information**

**1. Supplemental Text**

**1-1. Plasmid construction**

Individual single-guide RNA (sgRNA) expression vectors (pKLV-EF-LMNA-1L, -1R, -2L, and -2R for *lmna* targeting; pKLV-EF-LBR-1L, -1R, -2L, and -2R for *lbr* targeting) were constructed by means of pKLV-U6gRNA-EF(BbsI)-PGKpuro2ABFP (Addgene, Plasmid #62348) as described previously [54]. The list of sequences of the oligonucleotides used is given in Table S1. pBSKΔB-CBh-Cas9n-pA was constructed by standard PCR and standard cloning methods and deposited in Addgene (Plasmid # 82617).

To construct pBSK-Lmna-probe, a genomic region was amplified by PCR from C57BL6/J genomic DNA as a template using Lmna-F-primer and Lmna-R-primer (Table S2). The amplified DNA fragment was purified and digested with SacI and KpnI. The digested fragment was then subcloned into pBSK at SacI/KpnI sites.

To construct pBSK-Lbr-probe, a genomic region was amplified by PCR from C57BL6/J genomic DNA as a template using Lbr-F-primer and Lbr-R-primer. The amplified fragment was purified and digested with NotI and KpnI. The digested fragment was then subcloned into pBSK at NotI/KpnI sites.

pLR5-CBh-dCas9-mNenoGreen-IRES-Hyg, which is a dCas9-mNeonGreen [55] expression vector, was constructed by standard PCR and standard cloning methods. Nucleotide sequence of the plasmid is shown in Fig. S8.

To construct pLR5-U6-sgRNA-EF-MaSat1 and pLR5-U6-sgRNA-EF-MaSat3, sets of oligos for MaSat1 and MsSat3 (Table S1) were annealed and inserted into the BbsI site of pLR5-U6-sgRNA-EF (Addgene Plasmid # 82635).

**1-2. Immunofluorescence assays**

Immunostaining was performed on fixed cells (4% paraformaldehyde [PFA] in BBS [50 mM BES, 280 mM NaCl, 1.5 mM Na_2_HPO_4_⋅2H_2_O] with 1 mM CaCl_2_, for 15 min), washed, and blocked for 30 min in BBT-BSA buffer (BBS with 0.5% BSA, 0.1% Triton X-100, and 1 mM CaCl_2_). The cells were incubated with primary antibodies overnight at 4°C at the following dilutions: anti-LamA/C (1:100; H. Herrmann, H. Zentgraf, DKFZ), anti-LBR (1:100; M. Zwerger, H. Herrmann, DKFZ), anti-Lamin B1 (1:100; ab133741, Abcam, Cambridge, MA), anti-Nanog (1:500; MLC-51, eBioscience, San Diego, CA), anti-Oct4 (1:500; ab19857, Abcam), anti-Nestin (1:200; MAB353, EMD Millipore), anti-β3-tubulin (1:200; 4466S, Cell Signaling Technology, Beverly, MA), anti-H4K20me3 (1:500; ab9053, Abcam), anti-H3K4me3 (1:1000; MA304B, Clontech, Palo Alto, CA). The cells were washed and blocked in BBT-BSA and then incubated with Hoechst 33342 (1:1000, Life Technologies) and Alexa-conjugated secondary antibodies (1:500, Life Technologies). Images were acquired using an Olympus IX83 microscope (Olympus, Tokyo, Japan) with a CSU-W1 confocal unit (Yokogawa, Tokyo, Japan).

**1-3. Counting of chromocenters**

mESCs, NSCs, and cells differentiating into neurons at 6, 9, and 16 days postdifferentiation in 8µ-slides (ibidi) were fixed with 4% PFA, washed twice with PBS, then stained with Hoechst 33342. Images were acquired using an Olympus IX83 microscope (Olympus) with a CSU-W1 confocal unit (Yokogawa) and a 100× Olympus oil immersion objective of 1.40 NA. A total of 101 z-planes per site spanning 20 µm (z-step = 200 nm) were acquired. The nuclear volume and the number of CC clusters were estimated from Hoechst 33342 signals using ImarisCell (Bitmap, Zurich, Switzerland).

**1-4. Establishment of LBR revertant cells**

To establish LBR-expressing DKO cells, we introduced an LBR expression vector using the *piggyBac* system [56,57] as follows: 5 × 10^4^ DKO-4 and C57BL6/J mESCs were seeded in a gelatin-coated 24-well plate and cultured for 12 h. The cells were transfected with 50 ng of pLR5-CBh-dCas9-mNeonGreen-IRES-Hyg (Fig. S8), 75 ng of pLR5-U6-sgRNA-EF-MaSat1, 75 ng of pLR5-U6-sgRNA-EF-MaSat3, 50 ng of pCAG-hyPBase [54] and 100 ng of either pLR5-CAG-NLS-tdiRFP670-2A-LBR (LBR expression vector, Addgene Plasmid # 82620) or pLR5-CAG-NLS-tdiRFP670 (control vector, Addgene Plasmid # 82621) using Lipofectamine 2000 (Life Technologies). After 4 h, the medium containing Lipofectamine was replaced with a regular medium. At 4 days post-transfection, the cells were trypsinized and replated into a gelatin-coated 10-cm culture dish. After 7 days, we picked 16 colonies and selected tdiRFP670-expressing colonies. Next, the established clones were induced to differentiate into NSCs as described above. Then, the established NSCs were induced to differentiate into neurons. At 6 days postdifferentiation, the cells were fixed with 4% PFA and subjected to immunofluorescence staining with an anti-LBR antibody. The nuclei were stained with Hoechst 33342. Images were acquired using an Olympus IX83 microscope (Olympus) with a CSU-W1 confocal unit (Yokogawa) (Fig. S7).

**1-5. Live-cell imaging without drug treatment**

A total of 4 × 10^4^ C57BL6/J and DKO-4 NSCs were resuspended in a neural induction medium (N2B27 containing FGF) and plated onto a poly-ornithine/laminin-coated 8µ-slide (ibidi). At 3 days postdifferentiation, the medium was replaced with a fresh one containing 0.25 µM SiR-Hoechst (Spirochrome, Stein am Rhein, Switzerland). After 1-h incubation, the cells were subjected to live-cell imaging by means of an Olympus IX83 microscope with a CSU-W1 confocal unit and a 100× Olympus oil-immersion objective of 1.4 NA. Fluorescent images were captured using an iXon3 EMCCD camera (Andor, Ireland), equipped with a 637-nm laser, a stage top microscope incubator (5% CO_2_ at 37°C; Tokai Hit, Shizuoka, Japan), and an ASI MS-2000 piezo stage (Applied Scientific Instrumentation, Eugene, OR), and the data were analyzed in the Metamorph software (Molecular Devices Corp., Sunnyvale, CA): 61 z-planes per site, spanning 12 µm, were acquired at 1-min intervals for 1 h.

To obtain nuclear periphery kymographs, maximal-intensity projections of image stacks of differentiating DKO cells were thresholded using the ImageJ software (National Institutes of Health, Bethesda, MD). To eliminate the cell migration effect, the centers of mass of images were aligned. After that, edges of nuclei were identified using the “find edge” tool of ImageJ. Time series of edge-detected images were converted to a z-stack and reconstructed in Imaris (Bitmap, Zurich, Switzerland) to form a surface.

For longer imaging, the cells were prepared as described above. After 3 days postdifferentiation, SiR-Hoechst stained cells were subjected to live imaging by means of an Olympus IX83 microscope with a CSU-W1 confocal unit and a 60× Olympus oil-immersion objective of 1.42 NA. Forty-one z-planes per site, spanning 12 µm, were acquired at 30-min intervals for 44 h.

**1-6. Cytoskeletal drug treatment and live imaging**

A total of 4 × 10^4^ C57BL6/J and DKO-4 NSCs were resuspended in the neural induction medium and plated onto a poly-ornithine/laminin-coated 8µ-slide (ibidi). After 24 h, the medium was replaced with a fresh neural induction medium containing 20 µM ciliobrevin D, 0.5 µM paclitaxel, 10 nM nocodazole, 10 µM blebbistatin, 0.5 µM latrunculin A or DMSO. At 3 days post-differentiation, the medium was replaced with a fresh neural induction medium containing 0.25 µM SiR-Hoechst and either a drug or DMSO. After 1 h of incubation, the cells were subjected to live-cell imaging by means of an Olympus IX83 microscope with a CSU-W1 confocal unit and a 100× Olympus oil-immersion objective of 1.4 NA. Fluorescent images were captured using an iXon3 EMCCD camera equipped with a 637-nm laser, a stage-top microscope incubator (5% CO_2_ at 37°C; Tokai Hit), and an ASI MS-2000 piezo stage (Applied Scientific Instrumentation), and the data were analyzed in the Metamorph software; 61 z-planes per site, spanning 12 µm, were acquired at 1-min intervals for 1 h.

Projected nuclear area fluctuation analysis was performed as described before [20]. Briefly, the projected nuclear area was measured by thresholding a maximal-intensity projection of z-stacks of the nucleus. This projected area was then plotted as a function of time and fitted with third-order polynomial curves using Microsoft Excel (Microsoft, Redmond, CA). The residual values were divided by the value of the polynomial at each time point and multiplied by 100 to calculate normalized residual area fluctuations as percentages. Such fluctuation values were combined from multiple cells and time points for each condition to obtain a normal distribution. Standard deviation (σ) of such a distribution indicates the amplitude of area fluctuations. To determine whether the difference in σ between various conditions is statistically significant, the two-sample F-test for variance was performed in Excel. Differences with P values less than 0.01 were considered significant.

Acquired images were filtered with a one-pixel diameter Median filter using ImageJ. For ease of recognition of CC clusters, the stack was duplicated and one of them was subjected to background subtraction using an ImageJ plugin with a rolling ball radius of 5 pixels. Then, the two stacks were merged. The centers of mass of the nucleus and CC clusters were estimated from those stacks using ImarisCell (Bitmap).

The MSD was calculated as the average change in distance between the centers of mass of the nucleus and CC clusters across all possible combinations of time points separated by the lag time Δ*t*: [*d*(*t*) −*d*(*t* +Δ*t*)]^2^ [58].

**1-7. Actin fiber imaging**

DKO-4 NSCs were induced to differentiate into neuron as described above. At 9 days post-differentiation, cells were fixed by 4% PFA and washed twice with PBS. Cells were stained by Hoechst 33342 and Alexa Fluor 488 Phalloidin (Thermo Fischer Scientific, Waltham, MA) following the manufacturer’s protocol, and imaged as described above.

For live imaging of actin dynamics, a total of 4 × 10^4^ C57BL6/J and DKO-4 NSCs were resuspended in the neural induction medium and plated onto a poly-ornithine/laminin-coated 8µ-slide (ibidi). At 2 days post-differentiation, the medium was replaced with a fresh neural induction medium containing 50 µM SiR-Actin (Spirochrome). At 3 days post-differentiation, the medium was replaced with a fresh neural induction medium containing 50 µM SiR-Actin and 25 nM Vybrant DyeCycle Orange Stain (Thermo Fischer Scientific, Waltham, MA). After 1 h incubation, the cells were subjected to live-cell imaging by means of an Nikon Ti-2 microscope with a CSU-W1 confocal unit and a 100× Nikon oil-immersion objective of 1.49 NA. Fluorescent images were captured using an iXon Ultra EMCCD camera equipped with a 488-nm laser, a 637-nm laser, a stage-top microscope incubator (5% CO_2_ at 37°C; Tokai Hit), and the data were analyzed in the NIS-elements software; 77 z-planes per site, spanning 15 µm, were acquired at 15-min intervals for 3 h.

**1-8. *Ex vivo* live imaging**

Eyes were enucleated from 15-days-old C57BL/6 mice, and the neural retina was separated from the cornea, the sclera, the lens, the iris, the ciliary body and the pigmented epithelium in CO_2_-independent medium (Thermo Fischer Scientific, Waltham, MA) supplemented with 4 mM L-glutamine (retinal medium). The isolated retina was incubated with Hoechst 33342 at 20 μg/ml in retinal medium for 30 min, and then rinsed with retinal medium. The retina was placed in 1% agarose in retinal medium on a custom-made imaging chamber with the photoreceptor cell layer facing up (Fig. 4A). The cover glass was placed over the retina and sealed with silicon.

Two-photon imaging was performed with Nikon A1R-MP+ microscope (Nikon, Japan) equipped with a GaAsP type Non-Descanned Detector and a resonant scanner. Images were captured with a 25x objective lens (NA 1.10, Nikon) on InSight DeepSee Ti:sapphire laser (Spectra-Physics, USA) tuned to a wavelength of 730 nm. Timelapse analysis of image stacks consisting of over 20 optical sections with 0.2 μm Z-steps were acquired with 10-min interval over 3 hours. Each image was averaged 16 times. Photoreceptors were scanned from the top of the retina under the microscope and identified in the outermost layer. The kymograph analysis was conducted using the ImageJ software. The centers of mass of CC clusters were estimated from image stacks using Imaris (Bitmap).

**1-9. Statistical analysis**

All statistical analyses were performed with Microsoft Excel. Details of individual tests are outlined within each figure or figure legend, including number performed (n) and the reported error as standard error of the mean (SEM). All statistics are * p < 0.01; *** p < 10^-10^, and calculated by two-tailed Student’s t test or two-sample F-tests.

**References**

54. Ochiai H, Sugawara T, Yamamoto T. Simultaneous live imaging of the transcription and nuclear position of specific genes. Nucleic Acids Res. 2015;43: e127–e127. doi:10.1093/nar/gkv624

55. Shaner NC, Lambert GG, Chammas A, Ni Y, Cranfill PJ, Baird MA, et al. A bright monomeric green fluorescent protein derived from Branchiostoma lanceolatum. Nat Methods. 2013;10: 407–409. doi:10.1038/nmeth.2413

56. Yusa K, Rad R, Takeda J, Bradley A. Generation of transgene-free induced pluripotent mouse stem cells by the piggyBac transposon. Nat Methods. 2009;6: 363–369. doi:10.1038/nmeth.1323

57. Yusa K, Zhou L, Li MA, Bradley A, Craig NL. A hyperactive piggyBac transposase for mammalian applications. Proc Natl Acad Sci USA. 2011;108: 1531–1536. doi:10.1073/pnas.1008322108

58. Vazquez J, Belmont AS, Sedat JW. Multiple regimes of constrained chromosome motion are regulated in the interphase Drosophila nucleus. Curr Biol. 2001;11: 1227–1239.

59. Meister P, Gehlen LR, Varela E, Kalck V, Gasser SM. Visualizing yeast chromosomes and nuclear architecture. Meth Enzymol. Elsevier; 2010;470: 535–567. doi:10.1016/S0076-6879(10)70021-5

**2. Supplemental Figures, Tables and Materials**

Supplementary Movies S1-S7

Supplementary Figures S1-S14

Supplementary Tables S1-S3

**Movies S1 to S7**

**S1 Movie.** Live imaging of *Lbr/LamA* double-knockout (DKO) neuronal cells at 3 days postdifferentiation on a longer time scale, example 1. DKO neural stem cells (NSCs) were induced to differentiate into neurons. At 3 days postdifferentiation, the cells were stained with SiR-Hoechst and subjected to live imaging by confocal microscopy. Images were taken at 30-min intervals for 44 h. Z-series of 25 focal planes with a step size of 0.5 μm were acquired. The cells actively migrated during the imaging period. Thus, the nucleus was centered to make it easier to follow. The maximal projected image sequence shows dynamic nuclear deformation and chromocenter (CC) clustering. Scale bars: 10 µm.

**S2 Movie.** Live imaging of *Lbr/LamA* double-knockout (DKO) neuronal cells at 3 days postdifferentiation on a longer time scale, example 2. DKO neural stem cells (NSCs) were induced to differentiate into neurons. At 3 days postdifferentiation, the cells were stained with SiR-Hoechst and subjected to live imaging by confocal microscopy. Images were taken at 30-min intervals for 44 h. Z-series of 25 focal planes with a step size of 0.5 μm were acquired. Cells actively migrated during the imaging period. Thus, the nucleus was centered to make it easier to follow. The maximal projected image sequence shows dynamic nuclear deformation and chromocenter (CC) clustering. Scale bars: 10 µm.

**S3 Movie.** Live imaging of *Lbr/LamA* double-knockout (DKO) neuronal cells at 3 days postdifferentiation on a longer time scale, example 3. DKO neural stem cells (NSCs) were induced to differentiate into neurons. At 3 days postdifferentiation, the cells were stained with SiR-Hoechst and subjected to live imaging by confocal microscopy. Images were taken at 30-min intervals for 44 h. A Z-series of 25 focal planes with a step size of 0.5 μm were acquired. Cells actively migrated during the imaging period. Thus, the nucleus was centered to make it easier to follow. The maximum projected image sequence shows dynamic nuclear deformation and chromocenter (CC) clustering. Scale bars: 10 µm.

**S4 Movie.** Results of numerical simulation of chromocenter (CC) clustering by dynamic nuclear deformation. The period of simulation is approximately 33 h.

**S5 Movie.** Live imaging of actin dynamics in *Lbr/LamA* double-knockout (DKO) neuronal cells at 3 days postdifferentiation, example 1. DKO neural stem cells (NSCs) were induced to differentiate into neurons. At 3 days postdifferentiation, the cells were stained with SiR-Actin and Vybrant DyeCycle Orange Stain and subjected to live imaging by confocal microscopy. Images were taken at 15-min intervals for 3 h. A Z-series of 77 focal planes with a step size of 0.2 μm were acquired. The maximum projected image sequence shows dynamic actin movement. In this movie, three images are connected horizontally. Left, middle, and right side of images represent merged, nucleus (Vybrant DyeCycle Orange Stain), and actins (SiR-Actin), respectively. Scale bars: 10 µm.

**S6 Movie.** Live imaging of actin dynamics in *Lbr/LamA* double-knockout (DKO) neuronal cells at 3 days postdifferentiation, example 2. DKO neural stem cells (NSCs) were induced to differentiate into neurons. At 3 days postdifferentiation, the cells were stained with SiR-Actin and Vybrant DyeCycle Orange Stain and subjected to live imaging by confocal microscopy. Images were taken at 15-min intervals for 3 h. A Z-series of 77 focal planes with a step size of 0.2 μm were acquired. The maximum projected image sequence shows dynamic actin movement. In this movie, three images are connected horizontally. Left, middle, and right side of images represent merged, nucleus (Vybrant DyeCycle Orange Stain), and actins (SiR-Actin), respectively. Scale bars: 10 µm.

**S7 Movie.** *Ex vivo* live imaging of P15 mouse rod cells. Retinal tissue was excised from P15 mouse, stained with Hoechst 33342 and subjected to live imaging using two-photon microscopy. Images were taken at 10-min intervals for 3 h. Z-series of 103 focal planes with a step size of 0.2 μm were acquired. Center of mass in chromocenter (CC) clusters of some cells are represented by colored balls. Color coded lines represent trajectories of CC clusters.

**S8 Movie.** Dynamics deformation with affinity between heterochromatin and nuclear envelop.

**Figure S1 to S14**

**S1 Fig.** Establishment of DKO cell lines. **(A)** The strategy for targeted gene inactivation for establishment of the *Lbr*/*Lama* double knockout (DKO) cell lines is shown in the left panel. Grey and black boxes indicate untranslated regions and coding sequences, respectively. The right-hand panel shows schematic representations of target sequences of clustered regularly interspaced short palindromic repeat (CRISPR)/Cas9 nickase (Cas9n) used in this study. The target and protospacer adjacent motif (PAM) sequences are indicated with blue and red letters, respectively. **(B)** Immunofluorescence staining showing expression of LBR, LamA/C, and lamin B1 in wild-type (WT) and DKO cells in the left panel. We established four DKO cell lines. Scale bar: 10 µm. **(C)** Southern blot analyses showing a CRISPR-induced deletion in the DKO cell clones. An asterisk represents a nonspecific band. Some obscure bands are highlighted by arrows. The right-hand panel represents genomic structures of *Lama* and *Lbr*. Blue lines represent the Southern blot probe used in this study. **(D, E)** Sequence alignment of a genomic DNA region around CRISPR target sites of *Lama* and *Lbr* in clones DKO-4 and -36 and WT.

**S2 Fig.** Representative immunofluorescent staining of pluripotency markers, Nanog and Oct4, in WT and DKO-4 embryonic stem cells (ESCs), neural stem cells (NSCs), and neurons at 9 days postdifferentiation. Nuclei were stained with Hoechst 33342. Scale bar, 50 µm.

**S3 Fig.** Representative immunofluorescent staining of a neural stem cell marker, Nestin, in WT and DKO-4 embryonic stem cells (ESCs), neural stem cells (NSCs) and neurons at 9 days postdifferentiation. Nuclei were stained with Hoechst 33342. Scale bar, 50 µm.

**S4 Fig.** Representative immunofluorescence of a neuron marker, βTub III, in WT and DKO-4 embryonic stem cells (ESCs), neural stem cells (NSCs), and neurons at 9 days postdifferentiation. The nuclei were stained with Hoechst 33342. Scale bar, 50 µm.

**S5 Fig.** Representative immunofluorescence of H4K20me3 and H3K4me3 in WT and DKO-4 embryonic stem cells (ESCs), neural stem cells (NSCs), and neurons at 9 days postdifferentiation. Nuclei were stained with Hoechst 33342. We confirmed that localization characteristics of histone marks (H4K20me3 and H3K4me3) are qualitatively similar to those in mouse rod cells (6). Scale bar, 5 µm.

**S6 Fig.** Correlation between the number of chromocenter clusters and nuclear volume. Nuclear volumes and the numbers of chromocenter clusters in DKO-4 embryonic stem cells (ESCs), neural stem cells (NSCs), and neurons at 6, 9, and 16 days postdifferentiation were measured using Imaris and plotted. The number of chromocenter clusters and nuclear volume correlated (Spearman rho = 0.609, *p* = 5.0298e-58, *n*_ESC_ = 33; *n*_NSC_ = 181; *n*_d6_ = 117; *n*_d9_ = 80; *n*_d16_ = 139).

**S7 Fig.** LBR expression rescues conventional nuclear architecture in postmitotic DKO cells. We established an LBR-reverted DKO-4 ESC cell line (DKO-4+LBR). Then, DKO-4 and DKO-4+LBR ESCs were induced to differentiate into NSCs. The established NSCs were induced to differentiate into neurons. At 6 days postdifferentiation, the cells were fixed with 4% PFA and subjected to immunofluorescence staining using an anti-LBR antibody. Nuclei were stained with Hoechst 33342. Scale bar, 5 µm. LBR-reverted DKO cells did not show inverted nuclear architecture.

**S8 Fig.** Nucleotide sequence of pLR5-CBh-dCas9-mNenoGreen-IRES-Hyg.

**S9 Fig.** Model formulation. (**A**) An example of a color plot of subnuclear compartments. The imaging data for a rod cell from a 6-day-old mouse (6) and a simulation example image are shown in the upper and lower panel, respectively. (**B**, **C**) The conditions for heterochromatin fusion. (**D**) The sub-nulcear domain of a subnuclear compartment. The interface thickness of a phase-field function,$\delta$ , corresponds to the sub-nulcear domain region of the subnuclear compartment. (**E**) Representative simulation for 12 numbers of a chromocenter case.

**S10 Fig.** The effect of nuclear size. The effect of nuclear size on CC clustering is shown for the same level of a deformation degree. (**A**) The case that nuclear size is fixed. (**B**) The case when nuclear size is decreased by 20%. (**C**) Deformation degrees for panels (A) and (B).

**S11 Fig.** The number of chromocenter (CC) clusters during differentiation. This data is the same as Fig. 1C, but represented as histogram, so that reader can recognize that there is considerable variation in the number of CCs even in cells at 16 days post-differentiation.

**S12 Fig.** Projected nuclear area fluctuations in DKO cells treated with cytoskeletal drugs. A normalized histogram of combined projected nuclear area fluctuations for all the cells and all time points in DKO cells treated with DMSO, blebbistatin, ciliobrevin D, nocodazole, or paclitaxel is shown in the right-hand panel (see the Supplemental Experimental Procedures for details). Solid lines represent Gaussian fitting. Area fluctuations versus time plots for multiple cells are presented in the left panel. *n*_DMSO_ = 1464 from 24 cells; *n*_blebbistatin_ = 1403 from 23 cells; *n*_Latrunculin A_ = 1220 from 20 cells; *n*_ciliobrevin D_ = 488 from eight cells; *n*_nocodazole_ = 427 from seven cells; *n*_paclitaxel_ = 549 from nine cells.

**S13 Fig.** Ensemble- and time-averaged MSD between CC clusters and nuclear center of mass. Ensemble- and time-averaged MSD between CC clusters and nuclear center of mass in cells treated with DMSO, blebbistatin (bleb), nocodazole (noco), paclitaxel (pac), ciliobrevin (cilio) or latrunculin A (LatA) during the 1-h imaging period are shown on a linear scale (*n*_DMSO_ = 194 from 28 cells; *n*_bleb_ = 133 from 21 cells; *n*_cilio_ = 84 from 14 cells; *n*_noco_ = 66 from 12 cells; *n*_pac_ = 158 from 22 cells; *n*_Latrunculin A_ = 141 from 23 cells). All error bars represent the standard error of measurements. Every MSD curve almost reached a plateau in the imaging period, suggesting that diffusible space of CC clusters is a constraint. Using the value of the plateau, we calculated a volume of a spherical confinement of CC movement (53) and found that the volume of spherical confinement in cells treated with blebbistatin and Latrunculin A was approximately three-fold smaller in comparison with the others.

**S14 Fig.** Perinuclear actin cap structure was not observed in differentiating DKO cells. After 9 days post-differentiation of DKO NSCs, cells were fixed and stained with Alexa 488 phalloidin and Hoechst 33342. In these cells, perinuclear actin cap structure was not observed.

**Table S1 to S3**

**S1 Table.** A list of the sequences of the oligonucleotides for construction of sgRNA expression vectors used in this study.

**S2 Table.** A list of the nucleotide sequences of primers used in this study.

**S3 Table.** Details of simulation parameters. The details of representative dimensional/nondimensional parameters are given in the following Table, except for $\alpha_{0},\alpha_{V},\alpha_{v},\alpha_{v_{0}},\beta_{0},\beta_{\varphi},\beta_{\psi}, \beta_{\psi_{0}}$, which are the same in both nondimensional and dimensional systems, such that $\alpha_{0}=25/6$, $\alpha_{V}=10/6$, $\alpha_{v}=\alpha_{v_{0}}=40/3$, $\beta_{0}=5/3$,$\beta_{\psi}=1$, and $\beta_{\psi}=\beta_{\psi_{0}}=1/6$.
