# Supplementary figures and images for: Role of dynamic nuclear deformation on genomic architecture reorganization
Source: PLoS Comput Biol. 2019 Sep 11;15(9):e1007289. doi: 10.1371/journal.pcbi.1007289 (PMC6738595; doi:10.1371/journal.pcbi.1007289)

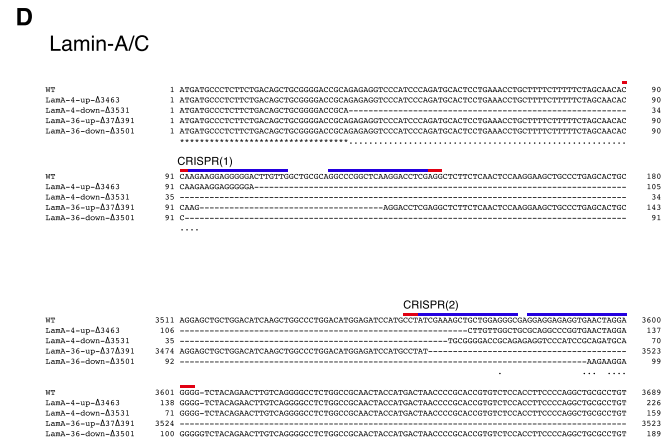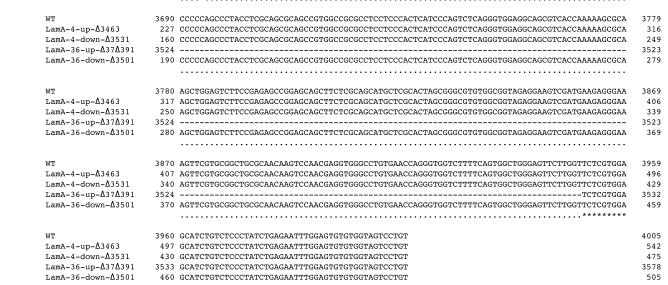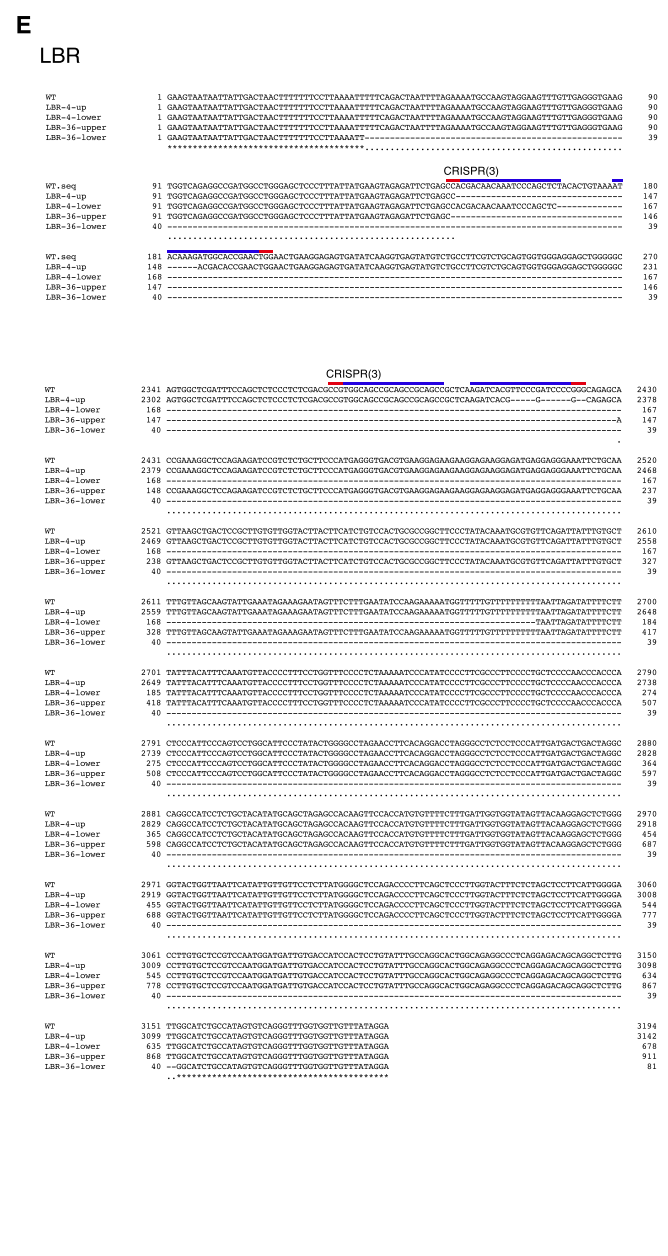

Supplement: S1 Fig — (A) The strategy for targeted gene inactivation for establishment of the Lbr/Lama double knockout (DKO) cell lines is shown in the left panel. Grey and black boxes indicate untranslated regions and coding sequences, respectively. The right-hand panel shows schematic representations of target sequences of clustered regularly interspaced short palindromic repeat (CRISPR)-Cas9 nickase (Cas9n) used in this study. The target and protospacer adjacent motif (PAM) sequences are indicated with blue and red letters, respectively. (B) Immunofluorescence staining showing expression of LBR, LamA/C, and lamin B1 in wild-type (WT) and DKO cells in the left panel. We established four DKO cell lines. Scale bar: 10 μm. (C) Southern blot analyses showing a CRISPR-induced deletion in the DKO cell clones. An asterisk represents a nonspecific band. Some obscure bands are highlighted by arrows. The right-hand panel represents genomic structures of Lama and Lbr. Blue lines represent the Southern blot probe used in this study. (D, E) Sequence alignment of a genomic DNA region around CRISPR target sites of Lama and Lbr in clones DKO-4 and -36 and WT. (PDF) [file pcbi.1007289.s010.pdf]

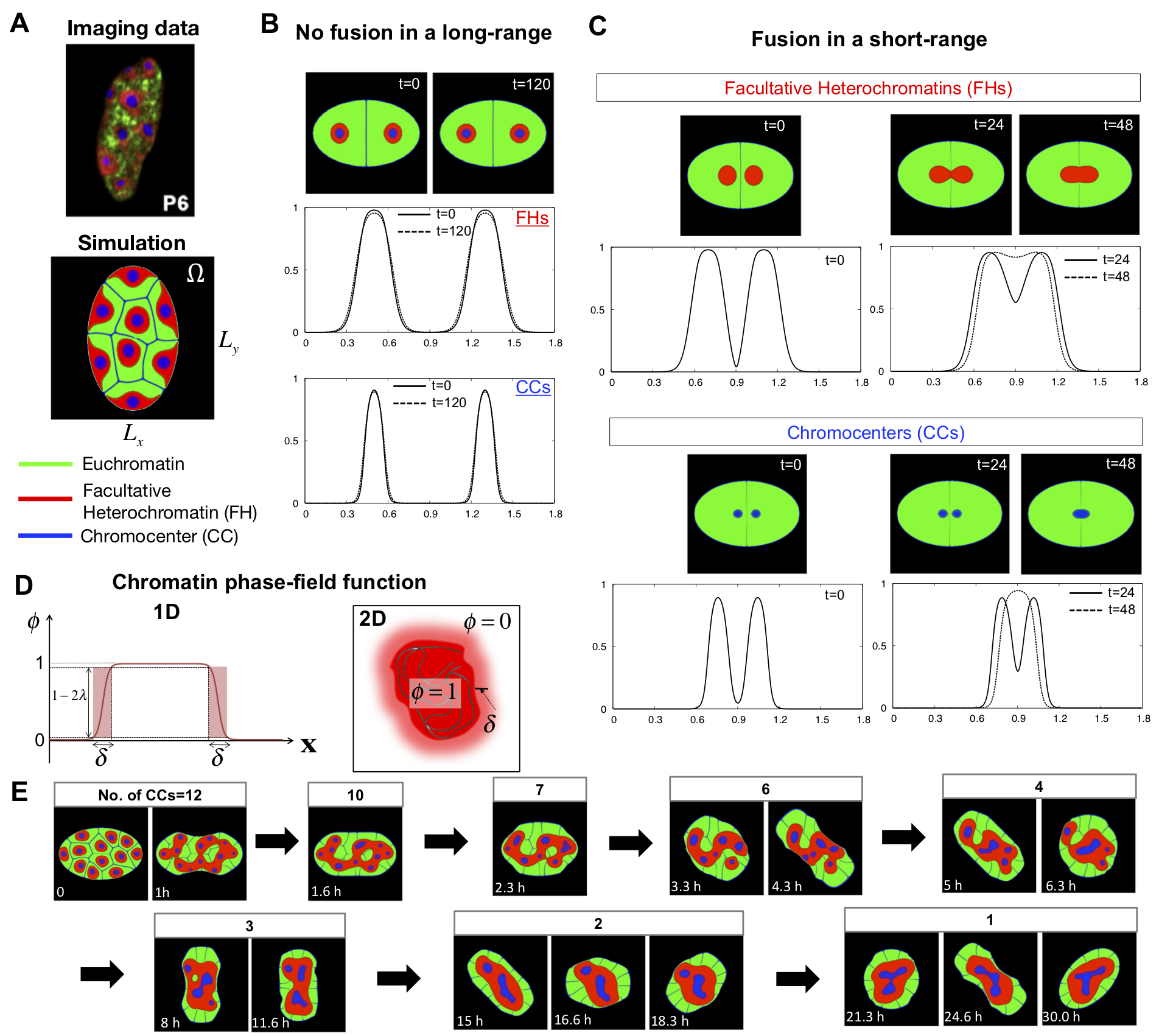

Supplement: S9 Fig — (A) An example of a color plot of subnuclear compartments. The imaging data for a rod cell from a 6-day-old mouse [6] and a simulation example image are shown in the upper and lower panel, respectively. (B, C) The conditions for heterochromatin fusion. (D) The sub-nulcear domain of a subnuclear compartment. The interface thickness of a phase-field function, δ, corresponds to the sub-nulcear domain region of the subnuclear compartment. (E) Representative simulation for 12 numbers of a chromocenter case. (TIFF) [file pcbi.1007289.s018.tiff]

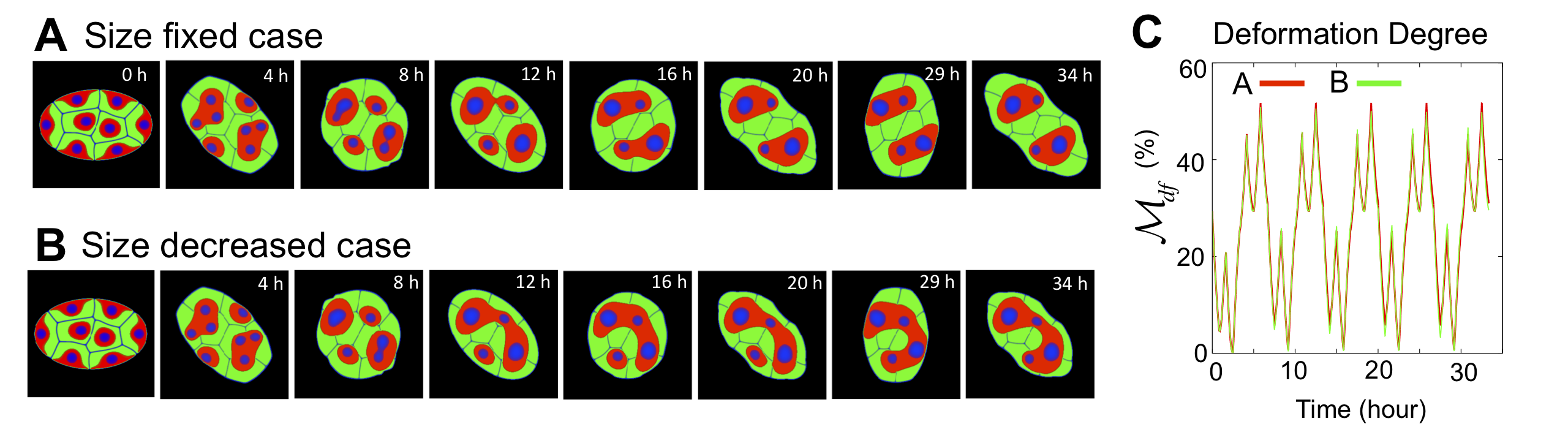

Supplement: S10 Fig — The effect of nuclear size on CC clustering is shown for the same level of a deformation degree. (A) The case that nuclear size is fixed. (B) The case when nuclear size is decreased by 20%. (C) Deformation degrees for panels (A) and (B). (TIFF) [file pcbi.1007289.s019.tiff]
