# Supplementary material for: Role of dynamic nuclear deformation on genomic architecture reorganization
Source: PLoS Comput Biol. 2019 Sep 11;15(9):e1007289. doi: 10.1371/journal.pcbi.1007289 (PMC6738595; doi:10.1371/journal.pcbi.1007289)
Supplement: S8 Fig — (PDF) [file pcbi.1007289.s017.pdf]

Supplementary Fig. S8, Nucleotide sequence of pLR5-CBh-dCas9-mNenoGreen-IRES-Hyg

```
CTAAATTGTAAGCGTTAATATTTTGTTAAAATTCGCGTTAAATTTTTGTTAAATCAGCTCATTTTTTAACCAATAGGCCGAAATCGGCAAAATCCCTTAT  < 100
        10        20        30        40        50        60        70        80        90

AAATCAAAAGAATAGACCGAGATAGGGTTGAGTGTTGTTCCAGTTTGGAACAAGAGTCCACTATTAAAGAACGTGGACTCCAACGTCAAAGGGCGAAAAA  < 200
       110       120       130       140       150       160       170       180       190

                                >F1 ori                                                <F1_origin
                                |                                                      |
CCGTCTATCAGGGCGATGGCCCACTACGTGAACCATCACCCTAATCAAGTTTTTTGGGGTCGAGGTGCCGTAAAGCACTAAATCGGAACCCTAAAGGGAG  < 300
       210       220       230       240       250       260       270       280       290

CCCCCGATTTAGAGCTTGACGGGGAAAGCCGGCGAACGTGGCGAGAAAGGAAGGGAAGAAAGCGAAAGGAGCGGGCGCTAGGGCGCTGGCAAGTGTAGCG  < 400
       310       320       330       340       350       360       370       380       390

                                                                                             <LacZ alpha
                                                                                             |
GTCACGCTGCGCGTAACCACCACACCCGCCGCGCTTAATGCGCCGCTACAGGGCGCGTCCCATTCGCCATTCAGGCTGCGCAACTGTTGGGAAGGGCGAT  < 500
       410       420       430       440       450       460       470       480       490

CGGTGCGGGCCTCTTCGCTATTACGCCAGCTGGCGAAAGGGGGATGTGCTGCAAGGCGATTAAGTTGGGTAACGCCAGGGTTTTCCCAGTCACGACGTTG  < 600
       510       520       530       540       550       560       570       580       590

                                                     PalAI
                                                     AscI
      >M13-fwd                   >T7                 SgsI
      |                          |                   |
TAAAACGACGGCCAGTGAGCGCGCGTAATACGACTCACTATAGGGCGAATTGGGCGCGCCATTCTAGATTAACCCTAGAAAGATAGTCTGCGTAAAATT  < 700
       610       620       630       640       650       660       670       680       690

GACGCATGCATTCTTGAAATATTGCTCTCTCTTTCTAAATAGCGCGAATCCGTCGCTGTGCATTTAGGACATCTCAGTCGCCGCTTGGAGCTCCCGTGAG  < 800
       710       720       730       740       750       760       770       780       790

                         >piggyBac 5 terminal repeat
                         |
GCGTGCTTGTCAATGCGGTAAGTGTCACTGATTTTGAACTATAACGACCGCGTGAGTCAAAATGACGCATGATTATCTTTTACGTGACTTTTAAGATTTA  < 900
       810       820       830       840       850       860       870       880       890

ACTCATACGATAATTATATTGTTATTTCATGTTCTACTTACGTGATAACTTATTATATATATATTTTCTTGTTATAGATATCAACTAGAATGCTAGCGTT  < 1000
       910       920       930       940       950       960       970       980       990

ACATAACTTACGGTAAATGGCCCGCCTGGCTGACCGCCCAACGACCCCCGCCCATTGACGTCAATAGTAACGCCAATAGGGACTTTCCATTGACGTCAAT  < 1100
      1010      1020      1030      1040      1050      1060      1070      1080      1090

                                  >CAG_enhancer
                                  |
GGGTGGAGTATTTACGGTAAACTGCCCACTTGGCAGTACATCAAGTGTATCATATGCCAAGTACGCCCCCTATTGACGTCAATGACGGTAAATGGCCCGC  < 1200
      1110      1120      1130      1140      1150      1160      1170      1180      1190

                                                       Eco105I
                                                       BstSNI
                                                       SnaBI
                                                       |
CTGGCATTGTGCCCAGTACATGACCTTATGGGACTTTCCTACTTGGCAGTACATCTACGTATTAGTCATCGCTATTACCATGGTCGAGGTGAGCCCCACG  < 1300
      1210      1220      1230      1240      1250      1260      1270      1280      1290

                                                                                               >CBh promoter
                                                                                               |
TTCTGCTTCACTCTCCCCATCTCCCCCCCCTCCCCACCCCCAATTTTGTATTTATTTATTTTTTAATTATTTTGTGCAGCGATGGGGGCGGGGGGGGGGG  < 1400
      1310      1320      1330      1340      1350      1360      1370      1380      1390

GGGGGCGCGCGCCAGGCGGGGCGGGGCGGGGCGAGGGGCGGGGCGGGGCGAGGCGGAGAGGTGCGGCGGCAGCCAATCAGAGCGGCGCGCTCCGAAAGTT  < 1500
      1410      1420      1430      1440      1450      1460      1470      1480      1490

TCCTTTTATGGCGAGGCGGCGGCGGCGGCGGCCCTATAAAAAGCGAAGCGCGCGGCGGGCGGGAGTCGCTGCGACGCTGCCTTCGCCCCGTGCCCCGCTC  < 1600
      1510      1520      1530      1540      1550      1560      1570      1580      1590

CGCCGCCGCCTCGCGCCGCCCGCCCCGGCTCTGACTGACCGCGTTACTCCCACAGGTGAGCGGGCGGGACGGCCCTTCTCCTCCGGGCTGTAATTAGCTG  < 1700
      1610      1620      1630      1640      1650      1660      1670      1680      1690

                                                                                               BshTI
                                                                                               CspAI
                                                                                               PinAI
                                                                                               AsiGI
                                                                                               AgeI
                                                                                               |
AGCAAGAGGTAAGGGTTTAAGGGATGGTTGGTTGGTGGGGTATTAATGTTTAATTACCTGGAGCACCTGCCTGAAATCACTTTTTTTCAGGTTGGACCGG  < 1800
      1710      1720      1730      1740      1750      1760      1770      1780      1790
```

Supplementary Fig. S8, continued

```
                        >HA tag                   >NLS
                        |                         |
TGCCACCATGTACCCATACGATGTTCCAGATTACGCTTCGCCGAAGAAAAAGCGCAAGGTCGAAGCGTCCGACAAGAAGTACAGCATCGGCCTGGCCATC  < 1900
         1810      1820      1830      1840      1850      1860      1870      1880      1890

GGCACCAACTCTGTGGGCTGGGCCGTGATCACCGACGAGTACAAGGTGCCCAGCAAGAAATTCAAGGTGCTGGGCAACACCGACCGGCACAGCATCAAGA  < 2000
         1910      1920      1930      1940      1950      1960      1970      1980      1990

AGAACCTGATCGGAGCCCTGCTGTTCGACAGCGGCGAAACAGCCGAGGCCACCCGGCTGAAGAGAACCGCCAGAAGAAGATACACCAGACGGAAGAACCG  < 2100
         2010      2020      2030      2040      2050      2060      2070      2080      2090

GATCTGCTATCTGCAAGAGATCTTCAGCAACGAGATGGCCAAGGTGGACGACAGCTTCTTCCACAGACTGGAAGAGTCCTTCCTGGTGGAAGAGGATAAG  < 2200
         2110      2120      2130      2140      2150      2160      2170      2180      2190

AAGCACGAGCGGCACCCCATCTTCGGCAACATCGTGGACGAGGTGGCCTACCACGAGAAGTACCCCACCATCTACCACCTGAGAAAGAAACTGGTGGACA  < 2300
         2210      2220      2230      2240      2250      2260      2270      2280      2290

GCACCGACAAGGCCGACCTGCGGCTGATCTATCTGGCCCTGGCCCACATGATCAAGTTCCGGGGCCACTTCCTGATCGAGGGCGACCTGAACCCCGACAA  < 2400
         2310      2320      2330      2340      2350      2360      2370      2380      2390

CAGCGACGTGGACAAGCTGTTCATCCAGCTGGTGCAGACCTACAACCAGCTGTTCGAGGAAAACCCCATCAACGCCAGCGGCGTGGACGCCAAGGCCATC  < 2500
         2410      2420      2430      2440      2450      2460      2470      2480      2490

CTGTCTGCCAGACTGAGCAAGAGCAGACGGCTGGAAAATCTGATCGCCCAGCTGCCCGGCGAGAAGAAGAATGGCCTGTTCGGCAACCTGATTGCCCTGA  < 2600
         2510      2520      2530      2540      2550      2560      2570      2580      2590

GCCTGGGCCTGACCCCCAACTTCAAGAGCAACTTCGACCTGGCCGAGGATGCCAAACTGCAGCTGAGCAAGGACACCTACGACGACGACCTGGACAACCT  < 2700
         2610      2620      2630      2640      2650      2660      2670      2680      2690

GCTGGCCCAGATCGGCGACCAGTACGCCGACCTGTTTCTGGCCGCCAAGAACCTGTCCGACGCCATCCTGCTGAGCGACATCCTGAGAGTGAACACCGAG  < 2800
         2710      2720      2730      2740      2750      2760      2770      2780      2790

ATCACCAAGGCCCCCCTGAGCGCCTCTATGATCAAGAGATACGACGAGCACCACCAGGACCTGACCCTGCTGAAAGCTCTCGTGCGGCAGCAGCTGCCTG  < 2900
         2810      2820      2830      2840      2850      2860      2870      2880      2890

AGAAGTACAAAGAGATTTTCTTCGACCAGAGCAAGAACGGCTACGCCGGCTACATTGACGGCGGAGCCAGCCAGGAAGAGTTCTACAAGTTCATCAAGCC  < 3000
         2910      2920      2930      2940      2950      2960      2970      2980      2990

CATCCTGGAAAAGATGGACGGCACCGAGGAACTGCTCGTGAAGCTGAACAGAGAGGACCTGCTGCGGAAGCAGCGGACCTTCGACAACGGCAGCATCCCC  < 3100
         3010      3020      3030      3040      3050      3060      3070      3080      3090

CACCAGATCCACCTGGGAGAGCTGCACGCCATTCTGCGGCGGCAGGAAGATTTTTACCCATTCCTGAAGGACAACCGGGAAAAGATCGAGAAGATCCTGA  < 3200
         3110      3120      3130      3140      3150      3160      3170      3180      3190

CCTTCCGCATCCCCTACTACGTGGGCCCTCTGGCCAGGGGAAACAGCAGATTCGCCTGGATGACCAGAAAGAGCGAGGAAACCATCACCCCCTGGAACTT  < 3300
         3210      3220      3230      3240      3250      3260      3270      3280      3290

CGAGGAAGTGGTGGACAAGGGCGCTTCCGCCCAGAGCTTCATCGAGCGGATGACCAACTTCGATAAGAACCTGCCCAACGAGAAGGTGCTGCCCAAGCAC  < 3400
         3310      3320      3330      3340      3350      3360      3370      3380      3390

AGCCTGCTGTACGAGTACTTCACCGTGTATAACGAGCTGACCAAAGTGAAATACGTGACCGAGGGAATGAGAAAGCCCGCCTTCCTGAGCGGCGAGCAGA  < 3500
         3410      3420      3430      3440      3450      3460      3470      3480      3490

AAAAGGCCATCGTGGACCTGCTGTTCAAGACCAACCGGAAAGTGACCGTGAAGCAGCTGAAAGAGGACTACTTCAAGAAAATCGAGTGCTTCGACTCCGT  < 3600
         3510      3520      3530      3540      3550      3560      3570      3580      3590

                                  PasI
                                  |
GGAAATCTCCGGCGTGGAAGATCGGTTCAACGCCTCCCTGGGCACATACCACGATCTGCTGAAAATTATCAAGGACAAGGACTTCCTGGACAATGAGGAA  < 3700
         3610      3620      3630      3640      3650      3660      3670      3680      3690

AACGAGGACATTCTGGAAGATATCGTGCTGACCCTGACACTGTTTGAGGACAGAGAGATGATCGAGGAACGGCTGAAAACCTATGCCCACCTGTTCGACG  < 3800
         3710      3720      3730      3740      3750      3760      3770      3780      3790
```

Supplementary Fig. S8, continued

```
ACAAAGTGATGAAGCAGCTGAAGCGGCGGAGATACACCGGCTGGGGCAGGCTGAGCCGGAAGCTGATCAACGGCATCCGGGACAAGCAGTCCGGCAAGAC  < 3900
         3810      3820      3830      3840      3850      3860      3870      3880      3890

                    >dCas9
                    |
AATCCTGGATTTCCTGAAGTCCGACGGCTTCGCCAACAGAAACTTCATGCAGCTGATCCACGACGACAGCCTGACCTTTAAAGAGGACATCCAGAAAGCC  < 4000
         3910      3920      3930      3940      3950      3960      3970      3980      3990

CAGGTGTCCGGCCAGGGCGATAGCCTGCACGAGCACATTGCCAATCTGGCCGGCAGCCCCGCCATTAAGAAGGGCATCCTGCAGACAGTGAAGGTGGTGG  < 4100
         4010      4020      4030      4040      4050      4060      4070      4080      4090

ACGAGCTCGTGAAAGTGATGGGCCGGCACAAGCCCGAGAACATCGTGATCGAAATGGCCAGAGAGAACCAGACCACCCAGAAGGGACAGAAGAACAGCCG  < 4200
         4110      4120      4130      4140      4150      4160      4170      4180      4190

CGAGAGAATGAAGCGGATCGAAGAGGGCATCAAAGAGCTGGGCAGCCAGATCCTGAAAGAACACCCCGTGGAAAACACCCAGCTGCAGAACGAGAAGCTG  < 4300
         4210      4220      4230      4240      4250      4260      4270      4280      4290

TACCTGTACTACCTGCAGAATGGGCGGGATATGTACGTGGACCAGGAACTGGACATCAACCGGCTGTCCGACTACGATGTGGACGCCATCGTGCCTCAGA  < 4400
         4310      4320      4330      4340      4350      4360      4370      4380      4390

GCTTTCTGAAGGACGACTCCATCGACAACAAGGTGCTGACCAGAAGCGACAAGAACCGGGGCAAGAGCGACAACGTGCCCTCCGAAGAGGTCGTGAAGAA  < 4500
         4410      4420      4430      4440      4450      4460      4470      4480      4490

GATGAAGAACTACTGGCGGCAGCTGCTGAACGCCAAGCTGATTACCCAGAGAAAGTTCGACAATCTGACCAAGGCCGAGAGAGGCGGCCTGAGCGAACTG  < 4600
         4510      4520      4530      4540      4550      4560      4570      4580      4590

GATAAGGCCGGCTTCATCAAGAGACAGCTGGTGGAAACCCGGCAGATCACAAAGCACGTGGCACAGATCCTGGACTCCCGGATGAACACTAAGTACGACG  < 4700
         4610      4620      4630      4640      4650      4660      4670      4680      4690

AGAATGACAAGCTGATCCGGGAAGTGAAAGTGATCACCCTGAAGTCCAAGCTGGTGTCCGATTTCCGGAAGGATTTCCAGTTTTACAAAGTGCGCGAGAT  < 4800
         4710      4720      4730      4740      4750      4760      4770      4780      4790

CAACAACTACCACCACGCCCACGACGCCTACCTGAACGCCGTCGTGGGAACCGCCCTGATCAAAAAGTACCCTAAGCTGGAAAGCGAGTTCGTGTACGGC  < 4900
         4810      4820      4830      4840      4850      4860      4870      4880      4890

GACTACAAGGTGTACGACGTGCGGAAGATGATCGCCAAGAGCGAGCAGGAAATCGGCAAGGCTACCGCCAAGTACTTCTTCTACAGCAACATCATGAACT  < 5000
         4910      4920      4930      4940      4950      4960      4970      4980      4990

TTTTCAAGACCGAGATTACCCTGGCCAACGGCGAGATCCGGAAGCGGCCTCTGATCGAGACAAACGGCGAAACCGGGGAGATCGTGTGGGATAAGGGCCG  < 5100
         5010      5020      5030      5040      5050      5060      5070      5080      5090

GGATTTTGCCACCGTGCGGAAAGTGCTGAGCATGCCCCAAGTGAATATCGTGAAAAAGACCGAGGTGCAGACAGGCGGCTTCAGCAAAGAGTCTATCCTG  < 5200
         5110      5120      5130      5140      5150      5160      5170      5180      5190

                                             SanDI
                                             KflI
                                             |
CCCAAGAGGAACAGCGATAAGCTGATCGCCAGAAAGAAGGACTGGGACCCTAAGAAGTACGGCGGCTTCGACAGCCCCACCGTGGCCTATTCTGTGCTGG  < 5300
         5210      5220      5230      5240      5250      5260      5270      5280      5290

TGGTGGCCAAAGTGGAAAAGGGCAAGTCCAAGAAACTGAAGAGTGTGAAAGAGCTGCTGGGGATCACCATCATGGAAAGAAGCAGCTTCGAGAAGAATCC  < 5400
         5310      5320      5330      5340      5350      5360      5370      5380      5390

CATCGACTTTCTGGAAGCCAAGGGCTACAAAGAAGTGAAAAAGGACCTGATCATCAAGCTGCCTAAGTACTCCCTGTTCGAGCTGGAAAACGGCCGGAAG  < 5500
         5410      5420      5430      5440      5450      5460      5470      5480      5490

AGAATGCTGGCCTCTGCCGGCGAACTGCAGAAGGGAAACGAACTGGCCCTGCCCTCCAAATATGTGAACTTCCTGTACCTGGCCAGCCACTATGAGAAGC  < 5600
         5510      5520      5530      5540      5550      5560      5570      5580      5590

TGAAGGGCTCCCCCGAGGATAATGAGCAGAAACAGCTGTTTGTGGAACAGCACAAGCACTACCTGGACGAGATCATCGAGCAGATCAGCGAGTTCTCCAA  < 5700
         5610      5620      5630      5640      5650      5660      5670      5680      5690

GAGAGTGATCCTGGCCGACGCTAATCTGGACAAAGTGCTGTCCGCCTACAACAAGCACCGGGATAAGCCCATCAGAGAGCAGGCCGAGAATATCATCCAC  < 5800
         5710      5720      5730      5740      5750      5760      5770      5780      5790
```

Supplementary Fig. S8, continued

```
CTGTTTACCCTGACCAATCTGGGAGCCCCTGCCGCCTTCAAGTACTTTGACACCACCATCGACCGGAAGAGGTACACCAGCACCAAAGAGGTGCTGGACG  < 5900
         5810      5820      5830      5840      5850      5860      5870      5880      5890

                                                                                    >NLS
                                                                                    |
CCACCCTGATCCACCAGAGCATCACCGGCCTGTACGAGACACGGATCGACCTGTCTCAGCTGGGAGGCGACAGCCCCAAGAAGAAGAGAAAGGTGGAGGC  < 6000
         5910      5920      5930      5940      5950      5960      5970      5980      5990

                                                                            BamHI
                                                                            |
CAGCCTCTCGAGGCCGGACCCCGCGCTGGCCGCACTCGGAGGCGGCGGAAGCGGTGGCGGAGGGTCAGGCGGAGGTGGATCCATGGTGAGTAAGGGAGAG  < 6100
         6010      6020      6030      6040      6050      6060      6070      6080      6090

GAAGATAATATGGCCTCCCTTCCCGCTACGCACGAACTCCACATCTTCGGGTCAATCAACGGTGTTGACTTCGACATGGTGGGCCAGGGCACCGGCAATC  < 6200
         6110      6120      6130      6140      6150      6160      6170      6180      6190

CCAATGACGGATACGAAGAACTCAATTTGAAGAGTACAAAGGGCGATCTCCAATTCTCACCTTGGATTCTGGTTCCCCACATTGGATACGGATTTCATCA  < 6300
         6210      6220      6230      6240      6250      6260      6270      6280      6290

                                                         BstEII
                                                         Eco065I
                                                         Eco91I
                                                         PspEI
                                                         BstPI
                                                         |
GTACCTGCCGTACCCCGATGGGATGAGCCCATTTCAGGCTGCAATGGTAGATGGTAGCGGTTACCAAGTACACCGAACTATGCAATTTGAGGACGGTGCC  < 6400
         6310      6320      6330      6340      6350      6360      6370      6380      6390

                                   >mNeonGreen
                                   |
TCACTGACAGTGAACTATCGGTATACTTACGAAGGAAGCCACATCAAGGGAGAGGCACAGGTCAAAGGAACCGGATTTCCAGCCGACGGGCCAGTCATGA  < 6500
         6410      6420      6430      6440      6450      6460      6470      6480      6490

                 >AlfI
                 |
CAAACTCCCTGACCGCCGCAGATTGGTGCCGCAGCAAAAAGACCTATCCAAATGACAAGACCATTATCTCGACATTCAAATGGAGCTACACCACCGGAAA  < 6600
         6510      6520      6530      6540      6550      6560      6570      6580      6590

CGGCAAACGCTATCGGTCTACCGCCAGGACAACCTACACATTTGCAAAACCTATGGCCGCAAACTATCTGAAAAACCAGCCGATGTATGTGTTCCGAAAG  < 6700
         6610      6620      6630      6640      6650      6660      6670      6680      6690

ACGGAATTAAAACACTCGAAAACAGAACTAAACTTTAAAGAGTGGCAGAAAGCCTTTACCGACGTAATGGGCATGGACGAGCTGTATAAGTGATCTAGAA  < 6800
         6710      6720      6730      6740      6750      6760      6770      6780      6790

                                        MssI
                                        PmeI  PspXI
                                        |     |
TGCTGATGGGCTAGCTAAGAATTCCTAGAGCTGATCCGCGTTTAAACTCGAGGTTAATTAATGAGCGGCCGCATAGATAACTGATCCAGTGTGCTGGAAT  < 6900
         6810      6820      6830      6840      6850      6860      6870      6880      6890

TAATTCGCTGTCTGCGAGGGCCAGCTGTTGGGGTGAGTACTCCCTCTCAAAAGCGGGCATGACTTCTGCGCTAAGATTGTCAGTTTCCAAAAACGAGGAG  < 7000
         6910      6920      6930      6940      6950      6960      6970      6980      6990

                                        >synth int
                                        |
GATTTGATATTCACCTGGCCCGCGGTGATGCCTTTGAGGGTGGCCGCGTCCATCTGGTCAGAAAAGACAATCTTTTTGTTGTCAAGCTTGAGGTGTGGCA  < 7100
         7010      7020      7030      7040      7050      7060      7070      7080      7090

                   OliI
                   AleI
                   |
GGCTTGAGATCTGGCCATACACTTGAGTGACAATGACATCCACTTTGCCTTTCTCTCCACAGGTGTCCACTCCCAGGTCCAACTGCAGGTCGAGCATGCA  < 7200
         7110      7120      7130      7140      7150      7160      7170      7180      7190

TCTAGGGCGGCCAATTCCGCCCCTCTCCCCCCCCCCCCTTTTCCCTCCCCCCCCCCTAACGTTACTGGCCGAAGCCGCTTGGAATAAGGCCGGTGTGCGTT  < 7300
         7210      7220      7230      7240      7250      7260      7270      7280      7290

                                                                                    AvrII
                                                                                    BlnI
                                                                                    AspA2I
                                                                                    XmaJI
                                                                                    |
TGTCTATATGTTATTTTCCACCATATTGCCGTCTTTTGGCAATGTGAGGGCCCGGAAACCTGGCCCTGTCTTCTTGACGAGCATTCCTAGGGGTCTTTCC  < 7400
         7310      7320      7330      7340      7350      7360      7370      7380      7390

CCTCTCGCCAAAGGAATGCAAGGTCTGTTGAATGTCGTGAAGGAAGCAGTTCCTCTGGAAGCTTCTTGAAGACAAACAACGTCTGTAGCGACCCTTTGCA  < 7500
         7410      7420      7430      7440      7450      7460      7470      7480      7490

                               >internal ribosome entry site IRES
                               |
GGCAGCGGAACCCCCCACCTGGCGACAGGTGCCTCTGCGGCCAAAAGCCACGTGTATAAGATACACCTGCAAAGGCGGCACAACCCCAGTGCCACGTTGT  < 7600
         7510      7520      7530      7540      7550      7560      7570      7580      7590
```

Supplementary Fig. S8, continued

```
                                                                                                KpnI
                                                                                                Acc65I
                                                                                                Asp718I
                                                                                                |
GAGTTGGATAGTTGTGGAAAGAGTCAAATGGCTCTCCTCAAGCGTATTCAACAAGGGGCTGAAGGATGCCCAGAAGGTACCCCATTGTATGGGATCTGAT  < 7700
         7610      7620      7630      7640      7650      7660      7670      7680      7690

CTGGGGCCTCGGTGCACATGCTTTACATGTGTTTAGTCGAGGTTAAAAAAACGTCTAGGCCCCCCGAACCACGGGGACGTGGTTTTCCTTTGAAAAACAC  < 7800
         7710      7720      7730      7740      7750      7760      7770      7780      7790

                    TspMI
                    SmaI                                                                BoxI
                    XmaI                                                                PshAI
                    Cfr9I                                                               BstPAI
                    |                                                                   |
GATGATAAGCTTGCCACAACCCGGGATAATTCCTGCAGCCAATATGAAAAAGCCTGAACTCACCGCGACGTCTGTCGAGAAGTTTCTGATCGAAAAGTTC  < 7900
         7810      7820      7830      7840      7850      7860      7870      7880      7890

     >Esp3I
     >BsmBI
     |
GACAGCGTCTCCGACCTGATGCAGCTCTCGGAGGGCGAAGAATCTCGTGCTTTCAGCTTCGATGTAGGAGGGCGTGGATATGTCCTGCGGGTAAATAGCT  < 8000
         7910      7920      7930      7940      7950      7960      7970      7980      7990

GCGCCGATGGTTTCTACAAAGATCGTTATGTTTATCGGCACTTTGCATCGGCCGCGCTCCCGATTCCGGAAGTGCTTGACATTGGGGAATTCAGCGAGAG  < 8100
         8010      8020      8030      8040      8050      8060      8070      8080      8090

CCTGACCTATTGCATCTCCCGCCGTGCACAGGGTGTCACGTTGCAAGACCTGCCTGAAACCGAACTGCCCGCTGTTCTGCAGCCGGTCGCGGAGGCCATG  < 8200
         8110      8120      8130      8140      8150      8160      8170      8180      8190

   RgaI                                            RsrII
   AsiSI                                           Rsr2I
   SfaAI                                           CpoI
   SgfI                                            CspI
   |                                               |
GATGCGATCGCTGCGGCCGATCTTAGCCAGACGAGCGGGTTCGGCCCATTCGGACCGCAAGGAATCGGTCAATACACTACATGGCGTGATTTCATATGCG  < 8300
         8210      8220      8230      8240      8250      8260      8270      8280      8290

                                                    >HygroR
                                                    |
CGATTGCTGATCCCCATGTGTATCACTGGCAAACTGTGATGGACGACACCGTCAGTGCGTCCGTCGCGCAGGCTCTCGATGAGCTGATGCTTTGGGCCGA  < 8400
         8310      8320      8330      8340      8350      8360      8370      8380      8390

GGACTGCCCCGAAGTCCGGCACCTCGTGCACGCGGATTTCGGCTCCAACAATGTCCTGACGGACAATGGCCGCATAACAGCGGTCATTGACTGGAGCGAG  < 8500
         8410      8420      8430      8440      8450      8460      8470      8480      8490

GCGATGTTCGGGGATTCCCAATACGAGGTCGCCAACATCTTCTTCTGGAGGCCGTGGTTGGCTTGTATGGAGCAGCAGACGCGCTACTTCGAGCGGAGGC  < 8600
         8510      8520      8530      8540      8550      8560      8570      8580      8590

ATCCGGAGCTTGCAGGATCGCCGCGGCTCCGGGCGTATATGCTCCGCATTGGTCTTGACCAACTCTATCAGAGCTTGGTTGACGGCAATTTCGATGATGC  < 8700
         8610      8620      8630      8640      8650      8660      8670      8680      8690

AGCTTGGGCGCAGGGTCGATGCGACGCAATCGTCCGATCCGGAGCCGGGACTGTCGGGCGTACACAAATCGCCCGCAGAAGCGCGGCCGTCTGGACCGAT  < 8800
         8710      8720      8730      8740      8750      8760      8770      8780      8790

GGCTGTGTAGAAGTACTCGCCGATAGTGGAAACCGACGCCCCAGCACTCGTCCGAGGGCAAAGGAATAGGGATCAATTCTCTAGATAACTGATCATAATC  < 8900
         8810      8820      8830      8840      8850      8860      8870      8880      8890

                                                                                   MunI        KspAI
                                                                                   MfeI        HpaI
                                                                                   |           |
AGCCATACCACATTTGTAGAGGTTTTACTTGCTTTAAAAAACCTCCCACACCTCCCCCTGAACCTGAAACATAAAATGAATGCAATTGTTGTTGTTAACT  < 9000
         8910      8920      8930      8940      8950      8960      8970      8980      8990

                                                   >SV40 early mRNA polyadenylation signal
                                                   |
TGTTTATTGCAGCTTATAATGGTTACAAATAAAGCAATAGCATCACAAATTTCACAAATAAAGCATTTTTTTCACTGCATTCTAGTTGTGGTTTGTCCAA  < 9100
         9010      9020      9030      9040      9050      9060      9070      9080      9090

                                                                                                SpeI
                                                                                                AhlI
                                                                                        SalI    BcuI
                                                                                        |       |
ACTCATCAATGTATCTTAACGCGTCGAGTGCATTCTAGTTGTGGTTTGTCCAAACTCATCAATGTATCTTATCATGTCTGTATACCGTCGACATACTAGT  < 9200
         9110      9120      9130      9140      9150      9160      9170      9180      9190

TAAAAGTTTTGTTACTTTATAGAAGAAATTTTGAGTTTTTGTTTTTTTTTAATAAATAAATAAACATAAATAAATTGTTTGTTGAATTTATTATTAGTAT  < 9300
         9210      9220      9230      9240      9250      9260      9270      9280      9290

                    >piggyBac 3 terminal repeat
                    |
GTAAGTGTAAATATAATAAAACTTAATATCTATTCAAATTAATAAATAAACCTCGATATACAGACCGATAAAACACATGCGTCAATTTTACGCATGATTA  < 9400
         9310      9320      9330      9340      9350      9360      9370      9380      9390
```

Supplementary Fig. S8, continued

```
         SplI
         Pfl23II
         PspLI
         BsiWI
         |
TCTTTAACGTACGTCACAATATGATTATCTTTCTAGGGTTAATCTAGTATACGCGTATGCGGCCGCTTAATTAATCCAGCTTTTGTTCCCTTTAGTGAGG  < 9500
         9410      9420      9430      9440      9450      9460      9470      9480      9490

                                  <M13-rev                    <LacO                         <lac
                                  |                           |                             |
GTTAATTGCGCGCTTGGCGTAATCATGGTCATAGCTGTTTCCTGTGTGAAATTGTTATCCGCTCACAATTCCACACAACATACGAGCCGGAAGCATAAAG  < 9600
         9510      9520      9530      9540      9550      9560      9570      9580      9590

TGTAAAGCCTGGGGTGCCTAATGAGTGAGCTAACTCACATTAATTGCGTTGCGCTCACTGCCCGCTTTCCAGTCGGGAAACCTGTCGTGCCAGCTGCATT  < 9700
         9610      9620      9630      9640      9650      9660      9670      9680      9690

                                             >LguI
                                             >SapI
                                             >BspQI
                                             >PciSI
                                             |
AATGAATCGGCCAACGCGCGGGGAGAGGCGGTTTGCGTATTGGGCGCTCTTCCGCTTCCTCGCTCACTGACTCGCTGCGCTCGGTCGTTCGGCTGCGGCG  < 9800
         9710      9720      9730      9740      9750      9760      9770      9780      9790

AGCGGTATCAGCTCACTCAAAGGCGGTAATACGGTTATCCACAGAATCAGGGGATAACGCAGGAAAGAACATGTGAGCAAAAGGCCAGCAAAAGGCCAGG  < 9900
         9810      9820      9830      9840      9850      9860      9870      9880      9890

AACCGTAAAAAGGCCGCGTTGCTGGCGTTTTTCCATAGGCTCCGCCCCCCTGACGAGCATCACAAAAATCGACGCTCAAGTCAGAGGTGGCGAAACCCGA  < 10000
         9910      9920      9930      9940      9950      9960      9970      9980      9990

CAGGACTATAAAGATACCAGGCGTTTCCCCCTGGAAGCTCCCTCGTGCGCTCTCCTGTTCCGACCCTGCCGCTTACCGGATACCTGTCCGCCTTTCTCCC  < 10100
         10010     10020     10030     10040     10050     10060     10070     10080     10090

TTCGGGAAGCGTGGCGCTTTCTCATAGCTCACGCTGTAGGTATCTCAGTTCGGTGTAGGTCGTTCGCTCCAAGCTGGGCTGTGTGCACGAACCCCCCGTT  < 10200
         10110     10120     10130     10140     10150     10160     10170     10180     10190

                         <ColE1 origin
                         |
CAGCCCGACCGCTGCGCCTTATCCGGTAACTATCGTCTTGAGTCCAACCCGGTAAGACACGACTTATCGCCACTGGCAGCAGCCACTGGTAACAGGATTA  < 10300
         10210     10220     10230     10240     10250     10260     10270     10280     10290

GCAGAGCGAGGTATGTAGGCGGTGCTACAGAGTTCTTGAAGTGGTGGCCTAACTACGGCTACACTAGAAGGACAGTATTTGGTATCTGCGCTCTGCTGAA  < 10400
         10310     10320     10330     10340     10350     10360     10370     10380     10390

GCCAGTTACCTTCGGAAAAAGAGTTGGTAGCTCTTGATCCGGCAAACAAACCACCGCTGGTAGCGGTGGTTTTTTTGTTTGCAAGCAGCAGATTACGCGC  < 10500
         10410     10420     10430     10440     10450     10460     10470     10480     10490

AGAAAAAAAGGATCTCAAGAAGATCCTTTGATCTTTTCTACGGGGTCTGACGCTCAGTGGAACGAAAACTCACGTTAAGGGATTTTGGTCATGAGATTAT  < 10600
         10510     10520     10530     10540     10550     10560     10570     10580     10590

CAAAAAGGATCTTCACCTAGATCCTTTTAAATTAAAAATGAAGTTTTAAATCAATCTAAAGTATATATGAGTAAACTTGGTCTGACAGTTACCAATGCTT  < 10700
         10610     10620     10630     10640     10650     10660     10670     10680     10690

AATCAGTGAGGCACCTATCTCAGCGATCTGTCTATTTCGTTCATCCATAGTTGCCTGACTCCCCGTCGTGTAGATAACTACGATACGGGAGGGCTTACCA  < 10800
         10710     10720     10730     10740     10750     10760     10770     10780     10790

                             <Eco31I
                             <Bso31I
                             <BsaI
                             |
TCTGGCCCCAGTGCTGCAATGATACCGCGAGACCCACGCTCACCGGCTCCAGATTTATCAGCAATAAACCAGCCAGCCGGAAGGGCCGAGCGCAGAAGTG  < 10900
         10810     10820     10830     10840     10850     10860     10870     10880     10890

GTCCTGCAACTTTATCCGCCTCCATCCAGTCTATTAATTGTTGCCGGGAAGCTAGAGTAAGTAGTTCGCCAGTTAATAGTTTGCGCAACGTTGTTGCCAT  < 11000
         10910     10920     10930     10940     10950     10960     10970     10980     10990

                     <AmpR
                     |
TGCTACAGGCATCGTGGTGTCACGCTCGTCGTTTGGTATGGCTTCATTCAGCTCCGGTTCCCAACGATCAAGGCGAGTTACATGATCCCCCATGTTGTGC  < 11100
         11010     11020     11030     11040     11050     11060     11070     11080     11090

                    <AmpR
                    |
AAAAAAGCGGTTAGCTCCTTCGGTCCTCCGATCGTTGTCAGAAGTAAGTTGGCCGCAGTGTTATCACTCATGGTTATGGCAGCACTGCATAATTCTCTTA  < 11200
         11110     11120     11130     11140     11150     11160     11170     11180     11190

CTGTCATGCCATCCGTAAGATGCTTTTCTGTGACTGGTGAGTACTCAACCAAGTCATTCTGAGAATAGTGTATGCGGCGACCGAGTTGCTCTTGCCCGGC  < 11300
         11210     11220     11230     11240     11250     11260     11270     11280     11290
```

Supplementary Fig. S8, continued

```
GTCAATACGGGATAATACCGCGCCACATAGCAGAACTTTAAAAGTGCTCATCATTGGAAAACGTTCTTCGGGGCGAAAACTCTCAAGGATCTTACCGCTG  < 11400
         11310      11320      11330      11340      11350      11360      11370      11380      11390

TTGAGATCCAGTTCGATGTAACCCACTCGTGCACCCAACTGATCTTCAGCATCTTTTACTTTCACCAGCGTTTCTGGGTGAGCAAAAACAGGAAGGCAAA  < 11500
         11410      11420      11430      11440      11450      11460      11470      11480      11490

ATGCCGCAAAAAAGGGAATAAGGGCGACACGGAAATGTTGAATACTCATACTCTTCCTTTTTCAATATTATTGAAGCATTTATCAGGGTTATTGTCTCAT  < 11600
         11510      11520      11530      11540      11550      11560      11570      11580      11590

    <Amp prom
     |
GAGCGGATACATATTTGAATGTATTTAGAAAAATAAACAAATAGGGGTTCCGCGCACATTTCCCCGAAAAGTGCCAC  < 11677
         11610      11620      11630      11640      11650      11660      11670
```

```
Features :
M13-fwd                                   : [599 : 616 - CW]
M13-rev                                   : [9544 : 9524 - CCW]
T7                                        : [626 : 645 - CW]
ColE1 origin                              : [10540 : 9912 - CCW]
F1 ori                                    : [13 : 453 - CW]
LacZ alpha                                : [528 : 460 - CCW]
LacO                                      : [9572 : 9550 - CCW]
AmpR                                      : [11351 : 10692 - CCW]
HygroR                                    : [7844 : 8869 - CW]
Amp prom                                  : [11619 : 11591 - CCW]
lac                                       : [9606 : 9577 - CCW]
synth int                                 : [6897 : 7192 - CW]
SV40 early mRNA polyadenylation signal    : [9027 : 9077 - CW]
NLS                                       : [1841 : 1861 - CW]
NLS                                       : [5975 : 5995 - CW]
mNeonGreen                                : [6083 : 6790 - CW]
dCas9                                     : [1871 : 5971 - CW]
AmpR                                      : [11549 : 10692 - CCW]
CBh promoter                              : [997 : 1795 - CW]
F1_origin                                 : [441 : 135 - CCW]
HA tag                                    : [1811 : 1837 - CW]
CAG_enhancer                              : [1001 : 1270 - CW]
piggyBac 5 terminal repeat                : [670 : 983 - CW]
piggyBac 3 terminal repeat                : [9201 : 9442 - CW]
internal ribosome entry site IRES         : [7241 : 7823 - CW]
```
