# Supplementary material for: Role of dynamic nuclear deformation on genomic architecture reorganization
Source: PLoS Comput Biol. 2019 Sep 11;15(9):e1007289. doi: 10.1371/journal.pcbi.1007289 (PMC6738595; doi:10.1371/journal.pcbi.1007289)
Supplement: S3 Table — The details of representative dimensional/nondimensional parameters are given in the following Table, except for α0, αV, αv, αv0, β0, βφ, βψ, βψ0, which are the same in both nondimensional and dimensional systems, such that α0 = 25/6, αV = 10/6, αv=αv0=40/3, β0 = 5/3, βψ = 1, and βψ=βψ0=1/6. (DOCX) [file pcbi.1007289.s026.docx]

| **Symbol** | **Dimensionless value** | **Symbol** | **Dimensional value** |
| --- | --- | --- | --- |
| $L_{ld}\times L_{sd}$ | 1.6 $\times1.0$ | $L_{ld}^{d}\times L_{sd}^{d}$ | $9.0\times5.625 ({\mu m}^{2})$ |
| $t$ | 1.0 | $T$ | $\left[ 1, 60 \right](m)$ |
| * | * | $\mu$ | $T^{-1} (h^{-1})$ |
| $\varepsilon_{\phi}^{2}, \varepsilon_{\psi}^{2}, \varepsilon_{\psi0}^{2}$ | $[1.0\times{10}^{-4}, 2.0\times{10}^{-4}$] | $\epsilon_{\phi}^{2}, \epsilon_{\psi}^{2}, \epsilon_{\psi0}^{2}$ | $\left[ 3.16\times{10}^{-3}, 6.32\times{10}^{-3} \right]({\mu m}^{2})$ |
| $\gamma$ | 0.0022/3 | $\gamma$ | 0.55 ${\mu m}^{2}$ |
